# Hippocampal lipidome and transcriptome profile alterations triggered by acute exposure of mice to GSM 1800 MHz mobile phone radiation: An exploratory study

**DOI:** 10.1002/brb3.1001

**Published:** 2018-05-22

**Authors:** Adamantia F. Fragopoulou, Alexandros Polyzos, Maria‐Despoina Papadopoulou, Anna Sansone, Areti K. Manta, Evangelos Balafas, Nikolaos Kostomitsopoulos, Aikaterini Skouroliakou, Chryssostomos Chatgilialoglu, Alexandros Georgakilas, Dimitrios J. Stravopodis, Carla Ferreri, Dimitris Thanos, Lukas H. Margaritis

**Affiliations:** ^1^ Department of Cell Biology and Biophysics Faculty of Biology University of Athens Zografou Athens Greece; ^2^ Department of Women’s and Children’s Health Karolinska Institutet Stockholm Sweden; ^3^ Institute of Molecular Biology, Genetics and Biotechnology Biomedical Research Foundation Academy of Athens Athens Greece; ^4^ Consiglio Nazionale delle Ricerche ISOF Bologna Italy; ^5^ Laboratory Animal Facilities Center of Clinical, Experimental Surgery and Translational Research Biomedical Research Foundation Academy of Athens Athens Greece; ^6^ Department of Biomedical Engineering University of West Attica Athens Greece; ^7^ Institute of Nanoscience and Nanotechnology (INN) NCSR Demokritos Athens Greece; ^8^ DNA Damage Laboratory Department of Physics School of Applied Mathematical and Physical Sciences National Technical University of Athens (NTUA) Athens Greece; ^9^ Present address: Joan and Sanford I. Weill Department of Medicine Weill Cornell Medical College New York 10065 New York

**Keywords:** brain, fatty acids, gene expression, membrane remodeling, radiofrequencies

## Abstract

**Background:**

The widespread use of wireless devices during the last decades is raising concerns about adverse health effects of the radiofrequency electromagnetic radiation (RF‐EMR) emitted from these devices. Recent research is focusing on unraveling the underlying mechanisms of RF‐EMR and potential cellular targets. The “omics” high‐throughput approaches are powerful tools to investigate the global effects of RF‐EMR on cellular physiology.

**Methods:**

In this work, C57BL/6 adult male mice were whole‐body exposed (n_E__xp_ = 8) for 2 hr to GSM 1800 MHz mobile phone radiation at an average electric field intensity range of 4.3–17.5 V/m or sham‐exposed (n_SE_ = 8), and the RF‐EMR effects on the hippocampal lipidome and transcriptome profiles were assessed 6 hr later.

**Results:**

The data analysis of the phospholipid fatty acid residues revealed that the levels of four fatty acids [16:0, 16:1 (6c + 7c), 18:1 9c, eicosapentaenoic acid omega‐3 (EPA, 20:5 ω3)] and the two fatty acid sums of saturated and monounsaturated fatty acids (SFA and MUFA) were significantly altered (*p *<* *0.05) in the exposed group. The observed changes indicate a membrane remodeling response of the tissue phospholipids after nonionizing radiation exposure, reducing SFA and EPA, while increasing MUFA residues. The microarray data analysis demonstrated that the expression of 178 genes changed significantly (*p *<* *0.05) between the two groups, revealing an impact on genes involved in critical biological processes, such as cell cycle, DNA replication and repair, cell death, cell signaling, nervous system development and function, immune system response, lipid metabolism, and carcinogenesis.

**Conclusions:**

This study provides preliminary evidence that mobile phone radiation induces hippocampal lipidome and transcriptome changes that may explain the brain proteome changes and memory deficits previously shown by our group.

## 1 INTRODUCTION

Mobile telephone technology has become prevalent the last decades, with 4.88 billion users recorded worldwide today and a forecast of 5.47 billion users in 2018 according to Statista (http://www.statista.com). Despite the benefits of these daily used devices, there is growing concern over their safety. The emitted radiofrequency electromagnetic radiation (RF‐EMR) is suspected to underlie several health issues, such as headaches, fatigue, behavioral problems, cognitive deficits, reduced fertility, and brain tumor development (Adams, Galloway, Mondal, Esteves, & Mathews, 2014; Carlberg & Hardell, 2014; Divan, Kheifets, Obel, & Olsen, 2008; Fragopoulou et al., 2010; Hardell & Carlberg, 2009), but the cellular effects of RF‐EMR still remain unknown. Oxidative stress and calcium dynamics imbalance seem to be the most predominant mechanisms, leading to membrane instability and gene and protein expression changes (Dasdag, Akdag, Ulukaya, Uzunlar, & Ocak, 2009; Fragopoulou et al., 2012; Minelli, Balduzzo, Milone, & Nofrate, 2007; Rao et al., 2008; Sokolovic et al., 2008; Yakymenko et al., 2016).

The brain is the organ mostly affected by this kind of radiation because of the very close proximity to the mobile phone during a call, which can last from seconds to hours per day. Hippocampus is integral to memory function and is a brain region greatly affected by mobile phone (MP) radiation. Several studies have documented mobile phone‐related learning and memory impairment in mice and rats (Fragopoulou et al., 2010; Hao et al., 2013; Li, Shi, Lu, Xu, & Liu, 2012; Narayanan, Kumar, Karun, Nayak, & Bhat, 2015; Narayanan, Kumar, Potu, Nayak, & Mailankot, 2009; Ntzouni, Skouroliakou, Kostomitsopoulos, & Margaritis, 2013; Ntzouni, Stamatakis, Stylianopoulou, & Margaritis, 2011), while others failed to provide any evidence connecting RF‐EMR with memory deficits (Dubreuil, Jay, & Edeline, 2003).

In 2001, it was proposed that proteomics and transcriptomics, as well as other high‐throughput screening techniques, could be the ideal approaches to identify molecular targets of mobile phone radiation, elucidate the health hazard issues, and unravel the most investigated but still unknown mechanism of electromagnetic fields (EMF) action (Leszczynski & Joenväärä, 2001). However, even 16 years later, only a few studies have focused on the impact of RF‐EMF on gene expression after large‐scale analysis [cf reviews (Fragopoulou & Margaritis, 2012; Leszczynski et al., 2012)]. Of these, only four are *in vivo* studies focused on the mammalian (mouse or rat) brain analyzing the whole‐genome transcriptome, with contradictory results (Belyaev et al., 2006; McNamee et al., 2016; Nittby, Widegren, et al., 2008; Paparini et al., 2008), and one analyzing the microRNA expression pattern (Zhao et al., 2014). In this study, we aimed to unravel potential responding genes or pathways in the whole transcriptome of mouse hippocampus after exposure to mobile phone radiation. It is important though to underline that the health effects of radiation result from a complex network of biological molecular interactions and reactivity, and it is advisable to have a multidisciplinary approach. In this view, we also addressed fatty acid‐based membrane lipidomics focusing on the role of fatty acids as structural and functional components of membrane phospholipids, with a specific distribution among saturated and unsaturated moieties depending on the tissue type, polyunsaturated fats being very important for nervous tissue (Ferreri & Chatgilialoglu, 2012). In the case of radiologically contaminated environment, such stress caused remodeling of the animal fatty acid pool of tissues *in vivo*, in particular involving an increase in unsaturated fatty acids as response to these conditions (Audette‐Stuart, Ferreri, Festarini, & Carr, 2012; Festarini, Sholtz, Stuart, Kim, & Ferreri, 2016). Concerning RF‐EMF to the best of our knowledge, there are no data of brain tissue lipidomic follow‐up before and after exposure to mobile phone radiation. Indirectly, researchers in the field highlighted the importance of the cellular membrane as a target for nonionizing EMF effects, irrespective of the order in which the EMF effects occur (Nittby, Grafstrom, et al., 2008; Nittby et al. 2009). Indeed, it was demonstrated that RF‐EMF can affect membrane functions through alterations on the calcium dynamics in neuronal stem cells (Rao et al., 2008).

This work is aiming to combine two omics approaches in order to unravel the underlying mechanisms of mobile phone radiation and potential cellular targets on the mouse hippocampal lipidome and transcriptome profiles that suggest further comprehensive combination with proteome changes and functional changes, such as memory deficits, previously reported by our group.

## 2 MATERIALS AND METHODS

### 2.1 Animals and housing

A total of 16 healthy 4‐ to 6‐week‐old C57BL/6 male mice were obtained from the Laboratory of Animal Facilities of the Biomedical Research Foundation of the Academy of Athens (BRFAA) and then transferred to our animal facility in the Department of Cell Biology and Biophysics of Athens University, where they were left for two weeks to get acclimatized.

Animal experimentation was performed in the animal facility of the Department of Cell Biology and Biophysics, and protocols were evaluated and approved by the Veterinary Service of the Prefecture of Athens (Permit Number 6237/17.10.12), as required by the Greek legal requirements for animal experimentation. All experiments were performed in accordance with the approved guidelines which are in agreement with the ethical recommendations of the European Communities Council Directive of 22 November 1986 (86/609/EEC) and with the ethical rules of the Bioethics Committee of the Faculty of Biology of Athens University. The facility in BFRAA is registered as a “breeding” and “experimental” facility, while the facility in the Department of Cell Biology and Biophysics is registered as an “experimental” facility according to the Greek Presidential Decree 56/2013, which harmonizes national legislation with the European Community Directive 63/2010 on the Protection of Animals Used for Scientific Purposes.

Mice were randomly assigned to two groups (*n *= 8/group): “exposed” (Exp) and “sham‐exposed” (SE). Each group was divided into two subgroups, which were subjected to the same manipulations but at different time periods (duplicates). Only male mice were used to be consistent with previous studies from our laboratory on dosimetry and behavioral paradigms (Fragopoulou et al., 2010, 2012). All mice were housed in groups of four in 1264C Eurostandard Type II open top Plexiglas cages [cages 267 mm (L) × 207 mm (W) × 140 mm (H) ‐floor area 370 cm^2^‐, Tecniplast, Milan, Italy] and were kept under standard laboratory conditions, at a room temperature of 22 ± 2°C, relative humidity of 40 ± 10%, and 12‐h light/dark cycle (lights on between 07:00–19:00 hr). All mice had *ad libitum* access to filtered tap water in drinking bottles and to pelleted chow (4RF21, Mucedola, Italy) that contained 12% water, 18.5% protein, 3% fat, 6% fiber, 7% ash, and 53.5% NFE (nitrogen‐free extract equivalent to the carbohydrate fraction of the diet), while the bedding in each cage (Scobis Uno, Mucedola, Italy) was autoclaved, nonallergenic, dust‐free, and NH_3_ absorbent. The cages were cleaned once a week, and at the same time, the animals were weighted. Taking into consideration the welfare of the animals, enrichment material was used within their home cages, that is, soft paper and small red plastic houses (The Mouse House, ACRE011, Tecniplast, Milan, Italy).

### 2.2 Exposure conditions

Animals were exposed or sham‐exposed within their home cages, free moving, as previously reported (Fragopoulou et al., 2010, 2012; Ntzouni et al., 2011, 2013). The “exposed” group mice were whole body irradiated for 2 hr continuously during the light cycle period (9–11 am), with a GSM 1800 MHz commercially available mobile phone (MP) in speaking mode (the mobile phone was in a “call” situation; a radio was playing in order to simulate human voice) placed 3 cm underneath the plastic cage housing the animals. The exposure setup (1 cage and 1 mobile phone) was placed inside a self‐constructed rectangular Faraday cage (“reflection chamber”) [43 cm (W) × 32 cm (D) × 32 cm (H)], which had the one side open in order to allow MP communication with the base station (see Fig. S1for a schematic representation of the exposure setup). The sham‐exposed mice were located at the other side of the room within an identical but closed from all sides Faraday “reflection chamber.” They were handled in the same way as the exposed mice, by the same experimenter, and were also exposed to the radio sound as the exposed mice. Therefore, exposed and sham‐exposed animals were under the same environmental conditions besides radiation.

### 2.3 Dosimetry

During the exposure period, electric field (E‐field) intensity of the RF mobile phone signal was measured using the NARDA SRM 3000 (NARDA Safety Test Solutions, Inc., Mönchengladbach, Germany) and a R&S FSL/6 spectrum analyzer (Rohde & Schwarz, Munich, Germany) connected to near‐field probes. Measurements of 6‐minute average and maximum E‐field strength were recorded in different positions inside the animal cage. The lowest average E‐field value within the cages was measured to be 4.3 V/m, while the highest average E‐field value was 17.5 V/m. Specific absorption rate (SAR) and the specific absorption energy (SAE) can approximately be calculated according to the equations SAR = σ*E* ^2^/ρ and SAE =  (σ*E* ^2^)/ρ * t, where *E* is the rms value of the averaged E‐field intensity, σ is the mean electrical conductivity of mouse brain tissue at 1880–1900 MHz, ρ is the mass density, and t is the time of the exposure (7200 s). According to Hasgall et al. (2015) and Peyman, Rezazadeh, & Gabriel (2001)*,* the average electric conductivity of mouse hippocampus at the aforementioned frequencies is 1.25/Sm and ρ is 1045 kg/m^3^. As mice were freely moving, they experienced the aforementioned range of E‐field values (4.3–17.5 V/m), and consequently, in our situation, brain SAR ranged from 0.022 to 0.366 W/kg and SAE ranged from 159.24 to 2637.56 J/kg. The worst‐case exposure scenario would be to consider the highest recorded average electric field intensity of 17.5 V/m in the animal cage and that the animals were continuously sitting at the position of the highest electric field strength, as also previously described (Stasinopoulou et al., 2016). Maximum instant electric field strength values recorded in the cage ranged from 50 to 120 V/m, but according to the ICRNIPR guidelines, the calculation of SAR by definition requires the E‐field average value. In any case, SAR values are highly approximate in describing the absorbed energy since the E‐field value within the tissue is not known and cannot be measured (Manta et al., 2017; Margaritis et al., 2014).

RF background signals in the exposure chambers were negligible. Extremely low frequency (ELF) signals were measured with the NARDA EFA 300 spectrum analyzer (NARDA Safety Test Solutions, Inc.) within the cages of the exposed animals during MP operation. Average values of 150 nT were measured in the frequency of 50 Hz, while ELF components of the time domain multiple access (TDMA) GSM signal consisting of 217 Hz and its harmonics were negligible.

Concerning the sham‐exposed animals, RF and ELF signals were negligible in their Faraday “reflection chamber.” No SAR value was then necessary to be calculated for sham exposure.

### 2.4 Hippocampus isolation

Six hours postexposure (in order to allow transcription to take place) or sham‐exposure, animals were sacrificed by cervical dislocation and hippocampus was isolated. Hippocampi from either the right or the left hemisphere (chosen in a random way) from all the animals were processed for lipidomics analysis, while the other half for transcriptomics analysis.

### 2.5 Lipidomics analysis

#### 2.5.1 Materials

Commercially available cis and trans fatty acid methyl esters (FAME) (Sigma‐Aldrich, Milan, Italy), dimethyl disulphide (Fluka, Milan, Italy), iodine (Carlo Erba, Milan, Italy), and 6 cis‐hexadecenoic acid methyl ester (Lipidox, Sweden) were used without further purification. Solvents such as chloroform, methanol, diethylether, and *n*‐hexane were HPLC grade and were purchased from Baker (Germany). Silica gel analytical thin‐layer chromatography (TLC) was performed on Merck silica gel 60 plates 0.25 mm thickness (Merck, Germany), and the spots were detected by spraying the plate with cerium ammonium sulfate/ammonium molybdate reagent (Fluka).

#### 2.5.2 Treatment of mouse hippocampus tissue samples

Each hippocampus per animal was immediately placed on ice after its removal, weighted, and then homogenized in 500 μl PBS. The tissue homogenates from the groups of sham‐exposed mice (n_SE_ = 8) and exposed mice (n_Exp_ = 8) were flushed with nitrogen gas and stored in −80°C until further processing. Hippocampi were not pooled per group for the lipidomics analysis.

#### 2.5.3 Lipid extraction

Tridistilled H_2_0 was added (1 ml) to the homogenates (ca 12 mg), and lipids were extracted with 2:1 chloroform/methanol (4 × 4 ml) according to the Folch method (Folch, Lees, & Sloane Stanley, 1957). The organic layers were dried on anhydrous Na_2_SO_4_ and evaporated to dryness. The weight of lipid extract was found to be 1.3–1.5 mg in all samples. TLC monitoring of SE and Exp samples (eluent: *n*‐hexane/Et_2_0 8:2) revealed that the extracts were mainly composed by phospholipids and cholesterol.

#### 2.5.4 Transesterification

In order to obtain fatty acid methyl esters (FAME) for analysis, the lipid extracts were treated with 1 ml of 0.5 M solution of KOH in methanol for 10 min at room temperature for quantitative conversion. The reaction mixture was quenched with brine (0.5 ml) and FAME were extracted with *n*‐hexane (3 × 2 ml), dried on anhydrous Na_2_SO_4_, and evaporated to dryness.

#### 2.5.5 GC analysis of FAME

The FAME mixture was dissolved in 10 μl of *n* –hexane and 1 μl was injected for GC analysis. FAME were analyzed by GC (Agilent 6850, Milan) equipped with a 60 m × 0.25 mm × 0.25 μm (50%‐cyanopropyl)‐methylpolysiloxane column (DB23, Agilent, USA), and a flame ionization detector with the following oven program: temperature started from 165°C, held for 3 min, followed by an increase of 1°C/min up to 195°C, held for 40 min, followed by a second increase of 10°C/min up to 240°C, and held for 10 min. A constant pressure mode (29 psi) was chosen with helium as carrier gas. Methyl esters were identified by comparison with the retention times of authentic samples.

#### 2.5.6 Dimethyldisulfide (DMDS) derivatization of FAME

In order to unambiguously resolve the chemical structures of hexadecenoic positional isomers, in particular delta 6 and delta 7 C16:1 isomers, the FAME mixture obtained from the previous steps in *n*‐hexane (50 μl) was treated with 50 μl of dimethyldisulfide (DMDS excess) and two drops of a 6% solution of iodine in diethyl ether. The reaction was stirred at room temperature for one night, and then, 5% aqueous solution of sodium thiosulfate (3 × 1 ml) was added. The organic layer was washed with brine (2 × 1 ml), dried on anhydrous Na_2_SO_4_, and evaporated to dryness. The DMDS FAME adducts were analyzed by GC/MS (Thermo Scientific Trace 1300) equipped with a 15 m × 0.25 mm × 0.25 μm TG‐SQC 5% phenyl methyl polysiloxane column, with helium as carrier gas, coupled to a mass selective detector (Thermo Scientific ISQ) with the following oven program: temperature started at 80°C, maintained for 2 min, increased at a rate of 15°C/min up to 140°C, increased at a rate of 5°C/min up to 280°C, and held for 10 min (Nichols, Volkman, & Everitt, 1989).

#### 2.5.7 Unsaturation and peroxidation indices calculation

The indices of unsaturation and peroxidation can be obtained by applying the following formulas: UI =  [(%monoenoic) + (%dienoic × 2)  +  (%trienoic × 3)  +  (%tetraenoic × 4)  +  (%pentaenoic × 5)  +  (%hexaenoic × 6)] and PI =  [(%monoenoic × 0.025)  +  (%dienoic × 1)  +  (%trienoic × 2)  +  (%tetraenoic × 4)  +  (%pentaenoic × 6)  +  (%hexaenoic × 8)] which have been already used by some of us to evidence the different lipid profiles of red blood cell membranes in offspring’s longevity (Puca et al., 2008).

#### 2.5.8 Statistical analysis

The IBM SPSS v.22 software (SPSS Inc., Chicago, IL, USA) (SPSS 22: http://scicrunch.org/resolver/SCR_002865) was used for the analysis of the lipidomics data. Results are presented as means ± SD. The nonparametric Mann–Whitney U test was applied to compare the two groups (SE vs. Exp), as the data were not normally distributed.

### 2.6 Microarray and quantitative real‐time PCR analysis

#### 2.6.1 RNA isolation and Affymetrix Genechip processing

Each hippocampus was immediately placed after its removal in a DNase/RNase‐free Eppendorf containing 500 μl of TRIzol reagent (TR‐118; MRC Inc., Cincinnati, OH, USA) and stored at −80°C until further processing. Samples were then defrosted and homogenized in TRIzol reagent, and total RNA was collected via aqueous/organic phase separation (chloroform treatment) and purified by a commercial RNA‐binding column (Nucleospin RNA isolation kit; Macherey‐Nagel, Düren, Germany). RNA integrity was evaluated using the Agilent 2100 Bioanalyzer (Agilent Technologies, USA) with an RNA Nano Chip kit (5067–1511) and was quantified by a Nanodrop 2000 spectrophotometer (NanoDrop Technologies, USA). Only high‐quality RNA (OD260/280 ≥ 2.0, RI*N *> 7.0) was used for the analysis of differential gene expression. Equal amounts of the four hippocampal total RNAs (from four mice each) per experimental condition (SE or Exp) were combined in one sample, and two RNA samples per experimental condition were prepared (2SE and 2Exp). Pooling of the hippocampal RNA preparations from different mice reduces intersample variability (Paparini et al., 2008). From this time on, sample processing was carried out by investigators unaware of sample origin.

The four pooled total RNA samples (2SE and 2Exp), 200 ng each, were processed for microarray analysis according to the Affymetrix standard protocols using the GeneChip^®^ 3’ IVT Plus Reagent (P/N 902415, Affymertix, USA). First‐strand cDNA containing a T7 promoter sequence was synthesized by reverse transcription, priming with T7 oligo(dT) primer. Second‐strand cDNA synthesis followed to produce a double‐stranded DNA template and simultaneously degrade RNA. The double stranded template was transcribed *in vitro* to synthesize biotinylated cRNA, which was purified using the 3’ IVT Amplification Kit Module 2. Its size distribution was evaluated using the Agilent Bioanalyzer, and its concentration was evaluated by Nanodrop spectrophotometer. Twelve (12) μg of biotinylated cRNA was fragmented and hybridized on Affymetrix Chips (Mouse Expression Array 430A 2.0 #900498) for 16 hr. Each chip comprised of 22600 probe sets representing transcripts and variants from over 14000 well‐characterized mouse genes that can be used to explore mechanisms behind biological and disease processes.

#### 2.6.2 Microarray data analysis

The raw CEL files were analyzed with the R statistical environment version 2.13 using the Bioconductor package. MAS5.0 algorithm was used for background correction and samples were median normalized. Presence/absence calls from MAS5.0 algorithm were used to classify probes/genes as expressed or nonexpressed. In order to discard probes representing the same transcript, we selected the ones with the highest intensity value across all samples. Welch t test (*p* value cutoff of 0.05) was applied in order to identify differentially expressed genes (DEGs). Clustering of DEGs was performed with Euclidean distance metric and average linkage in MeV (MultiExperiment Viewer) software. The microarray data were submitted to Gene Expression Omnibus (GEO) repository under the accession code GSE93199.

#### 2.6.3 Functional annotation and pathway analysis

Gene ontology annotation, pathway enrichment, transcription factor regulation analysis, and network analysis were performed with the use of DAVID knowledgebase (Huang da, Sherman, & Lempicki, 2009a,2009b) and Ingenuity Pathway Analysis (IPA) software.

#### 2.6.4 Quantitative real‐time polymerase chain reaction (qRT‐PCR)

To confirm microarray gene expression profiles, oligonucleotides were designed for selected MP radiation‐modulated genes and qRT‐PCR was performed. The total RNAs of the four samples that were used in Affymetrix experiments were normalized, and further DNA digestion was carried out for 1 hr at 37°C using HaeIII (New England Biolabs, USA) and DNase I (New England Biolabs). Afterwards, cDNAs were produced employing the RevertAid^™^ H Minus Reverse Transcriptase (EP0451, Fermentas Lifesciences), OligodT primmer (5’ T(21) 3’ IDT), and dNTP Mix (Invitrogen #10297 018).

Primer design was carried out by the IDT primer quest software and corrected manually using the help of the appropriate Web sites (http://www.operon.com; Oligocalc; NCBI nucleotide BLAST). Primers were used at a concentration of 0.35 μΜ. Real‐time PCR amplification, followed by melting curve analysis, was carried out in 15 μl reaction volume with the Bio‐Rad CFX 96 Real Time System and Maxima SYBR Green qPCR Master Mix (K0259; Thermo Scientific). GAPDH was used as murine endogenous control. Samples were duplicated, and qRT‐PCR was repeated three times for every gene.

Welch t test was used for the analysis of the data using the IBM SPSS v22 software. Differences with *p* value ≤0.05 were considered significant. Sequences for forward and reverse primers as well as the amplicon size are shown in Table S1.

## 3 RESULTS

### 3.1 Lipidome profile changes in hippocampal tissue

#### 3.1.1 Fatty acid analysis of hippocampus homogenates

The hippocampus homogenates were worked up as described in the Materials and Methods section. It is worth mentioning that lipid extracts were made of phospholipids and cholesterol, which are the lipid constituents of hippocampus cell membranes. The fatty acid composition of phospholipids was determined after gas chromatography (GC) analyses of the corresponding FAME. GC was settled with a protocol that separates geometrical (cis and trans) and positional isomers, in particular for C16 monounsaturated fatty acids as previously described (Sansone, Melchiorre, Chatgilialoglu, & Ferreri, 2013). In the latter case, we were able to distinguish the three main isomers of C16 monounsaturated fatty acids (MUFA), namely palmitoleic acid (9*cis*‐16:1), sapienic acid (6*cis*‐16:1) and 7*cis*‐16:1 (Clarke, 2000; Shinitzky, 1984) (see Figs S2 and S3 for a representative GC analysis).

**Figure 1:**
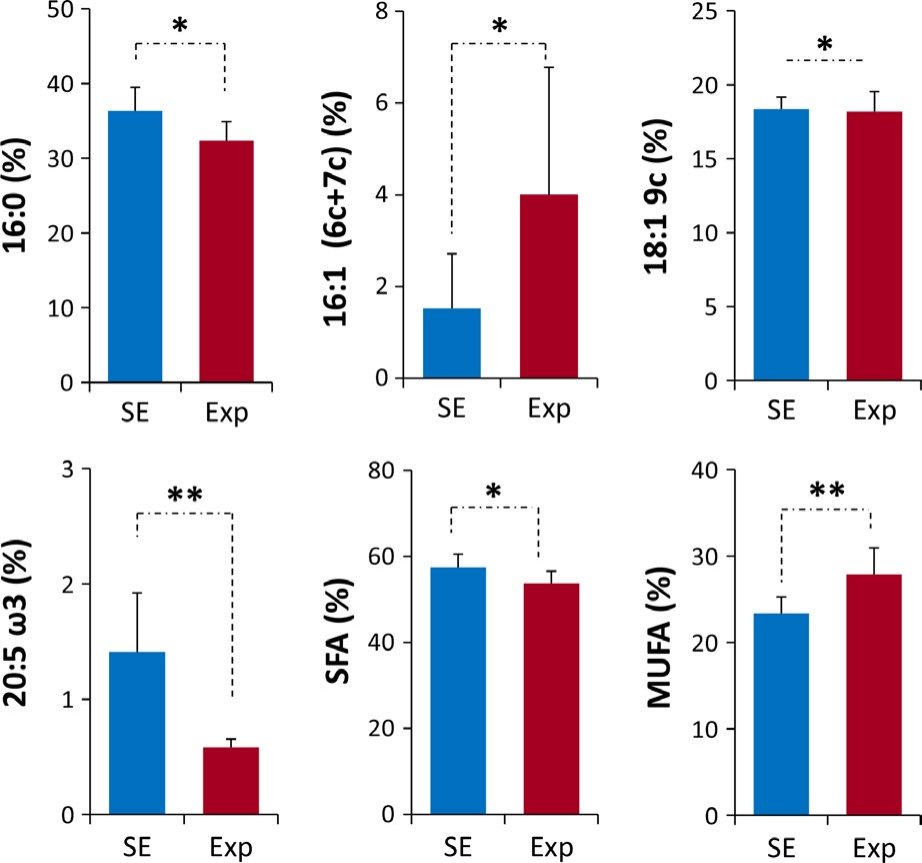
The graph depicts the fatty acid residues found in the hippocampus tissues of the exposed (Exp) and the sham‐exposed (SE) mice. Values are expressed as % rel (as reported in Table 1), and only, the statistically significant different fatty acids are reported. *p ≤* *0.05, **p ≤* *0.01

Table 1 reports the values corresponding to the total fatty acid composition in hippocampal tissues of untreated (SE) and treated (Exp) mice, expressed as relative percentages. The significant fatty acid changes (% rel of Table 1) are depicted in the graphics of Figure 1. The results depict statistically significant differences between the two groups. In particular, the changes included: a) the decrease of C16:0 (saturated fatty acids, SFA, palmitic acid (*p *=* *0.021), b) the increase of C16 MUFA (6cis‐16:1 and 7cis‐16:1; *p *=* *0.038) and C18:1 MUFA 9cis‐18:1 (oleic acid; *p *=* *0.028), c) the decrease of 20:5 ω3 (eicosapentaenoic acid EPA, *p *=* *0.004). The decrease in SFA and increase in MUFA are also significant where the sum of the fatty acids forming the same family was taken into account (*p *=* *0.028 and *p *=* *0.01, respectively). Importantly, examining the position of the double bond in the C16 MUFA, using a protocol described in human blood samples (Sansone et al., 2016), we found that the C16 MUFA with the double bond in position 6 (6*cis*‐16:1, sapienic acid) was selectively increased. Concerning the unsaturation and peroxidation indices’ values, there was no significant variation between the two groups of mice (*p *>* *0.05) (Table 2). Evaluating the behavior of the PUFA fatty acid contents, the mobile phone radiation exposure did not cause global changes of this type of fatty acid residues, but instead, it affected some of them. While only a trend of omega‐6 fatty acid diminution (linoleic, dihomo gammalinolenic, and arachidonic acids) could be observed, no changes were detected in the omega‐3 fatty acid alpha‐linolenic acid (C18:3), docosapentaenoic acid (DPA, C22:5), or docosahexaenoic acid (DHA, C22:6). Interestingly, the only significant PUFA that diminished was the omega‐3 eicosapentaenoic acid (EPA, 20:5) (*p *=* *0.004).

**Table 1:** Fatty acid methyl esters (FAME) (means ± SD) expressed as relative percentages (% rel) based on the total fatty acid peaks appeared in the gas chromatography (GC) analysis with >98% resolved fatty acid (FA) peaks/as detected by GC analysis of the lipid extracts obtained from the hippocampus homogenates of sham‐exposed (SE) and exposed (Exp) mice (see Fig. S2 for a representative GC analysis)

FAME	SE (*n *= 8)	Exp (*n *= 8)
	Mean ± SD (% rel)	Mean ± SD (% rel)
14:0	1.1 ± 0.3	1.3 ± 0.9
16:0	**36.3** ± **3.2**	**32.3** a ± **2.5**
16:1 (6c+7c)†	**1.5** ± **1.2**	**4.0** b ± **2.8**
16:1 9c	1.6 ± 0.6	1.5 ± 0.3
18:0	20.0 ± 2.6	18.3 ± 3.2
18:1 9c	**16.3** ± **0.8**	**18.2** c ± **1.4**
18:1 11c	3.3 ± 0.6	3.7 ± 1.0
18:2 ω6	3.9 ± 2.4	2.8 ± 1.1
18:3 ω3	0.7 ± 0.6	0.6 ± 0.2
20:3 ω6	0.9 ± 0.4	0.8 ± 0.2
20:4 ω6	4.8 ± 2.2	5.2 ± 0.8
20:5 ω3	**1.4 ± 0.5**	**0.6** d ± **0.1**
22:5 ω3	0.5 ± 0.1	0.5 ± 0.1
22:6 ω3	6.5 ± 2.8	7.1 ± 3.0
SFA	**57.4 ± 3.1**	**53.8** e ± **2.8**
MUFA	**23.4 ± 1.9**	**27.9** f ± **3.0**
PUFA	18.8 ± 2.0	18.2 ± 1.9
TOT trans‡	0.4 ± 0.2	0.4 ± 0.1

FAME, fatty acid methyl esters; SE, sham‐exposed; SFA, saturated fatty acids (SFA); MUFA, monounsaturated fatty acids. The bold character in the table indicates the fatty acid values with statistical significance.

**†**Sum of positional isomers 16:1 6 cis + 16:1 7 cis. The two positional isomers overlapped in only one peak in our GC conditions; we determined the presence of both positional isomers by recognition of diagnostic fragmentations after GC‐MS injection of their dimethyldisulfide (DMDS) adducts (Nichols et al., 1989). ‡TOT trans: the sum of geometrical trans isomers as determined by the analytical protocol previously described (Sansone et al., 2016).

^a^ *p* = 0.021, ^b^ *p* = 0.038, ^c^ *p* = 0.028, ^d^ *p* = 0.004, ^e^ *p* = 0.028, ^f^ *p* = 0.01.

**Table 2:** Unsaturation Index (UI) and Peroxidation Index (PI) obtained from the data of Table 1 according to known equations for the calculated values (Puca et al., 2008). Data are shown as means ± SD. SE: Sham‐exposed mice; Exp = exposed to mobile phone radiation mice

SE	Exp
UI	
98.9 ± 20.1	102.3 ± 18.3
PI	
89.3 ± 25.2	89.5 ± 25.6

SE, sham‐exposed; Exp, exposed.

### 3.2 Transcriptome profile changes in hippocampal tissue

#### 3.2.1 Identification of differentially expressed hippocampal transcripts in mobile phone exposed mice

**Figure 2:**
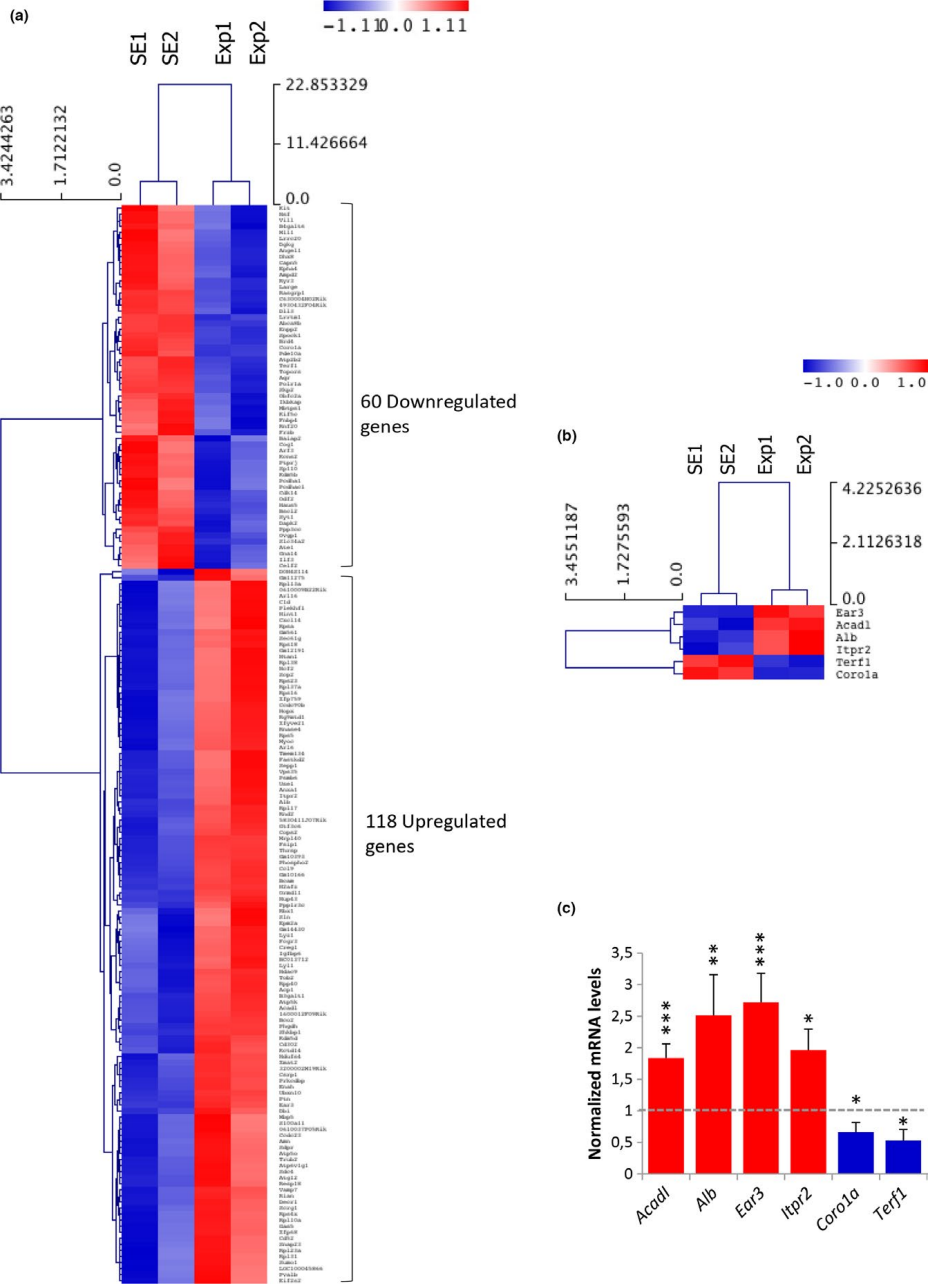
(a) Heat‐map showing normalized expression levels of 178 identified DEGs (≥1.5 fold, *p *≤* *0.05, Welch’s t test). Mobile phone radiation alters gene expression profile in mouse hippocampus mainly through a transcriptional activation process. Gene clustering was performed using Euclidean distance and average linkage analysis software. Red color indicates upregulated genes (118), while blue color specifies downregulated genes (60). SE: Sham‐exposed. Exp: Exposed. SE1, SE2, Exp1, and Exp2 refer to four pooled hippocampi each from four different mice. (b) Heat map of the six DEGs that were selected from the microarray experiment for qRT‐PCR verification. (c) Bar graph presenting average normalized mRNA levels ± SD (Exp1 and Exp2 vs. SE1 and SE2). *p ≤* *0.05, **p ≤* *0.01, ***p ≤* *0.001

To elucidate differences in hippocampal gene expression of mobile phone exposed and sham‐exposed mice, Affymetrix Mouse 430A 2.0 expression arrays were utilized. The mean expression of transcripts from the hippocampi of two sham‐exposed samples was compared to the expression of two exposed samples using the Welch t test. Genes with fold change ≥1.5 and *p* value ≤0.05 were considered as differentially expressed. This approach led to the identification of 178 transcripts modulated by mobile phone radiation exposure. More specifically, 118 genes were significantly activated (1.50–43.64 fold), while 60 genes were repressed (0.66–0.03 fold) under the specific exposure conditions (Table S2). Importantly, the majority of hippocampal genes identified herein were upregulated rather than downregulated after RF exposure (Figure 2a), suggesting that the primary genomewide response of the hippocampus to mobile phone radiation is gene activation.

Six genes were selected for further verification of the microarray analysis (relevant heat map shown in Figure 2b) by qRT‐PCR. Genes that were classified in different biological processes were selected. In particular, *Acadl* (acyl‐Coenzyme A dehydrogenase, long chain) was related to lipid metabolism and fatty acid beta‐oxidation; *Alb* (Albumin) to lipid metabolism, neurological diseases, and inflammatory response; *Ear3* (eosinophil‐associated, ribonuclease A family, member 3) to immune response (mediating chemotactic activity of dendritic cells); *Itpr2* (inositol 1,4,5‐triphosphate receptor 2) to cell signaling, calcium signaling, carbohydrate metabolism, synaptic long‐term potentiation (LTP), and neurological diseases; *Coro1a* (coronin, actin binding protein 1A) to immunity and mitochondrial apoptosis, and *Terf1* (telomeric repeat binding factor 1) to cell cycle and cancer. The qRT‐PCR analysis confirmed the expression profile for all six candidate genes (Figure 2c). *Alb* and *Ear3* showed more than 2.5‐fold transcriptional induction in response to RF radiation (*p *=* *0.003 and *p *<* *0.001, respectively), *Acadl* and *Itpr2* were upregulated 1.83‐fold (*p *=* *0.001) and 1.96‐fold (*p *=* *0.017), respectively, while *Coro1a* and *Terf1* were both characterized by a significant reduction of their gene expression capacities (1.5‐fold and 1.89‐fold, *p *=* *0.017, and *p *=* *0.049, respectively) under the same exposure conditions.

#### 3.2.2 Functional categorization of mobile phone radiation‐specific differentially expressed genes (DEGs)

**Figure 3:**
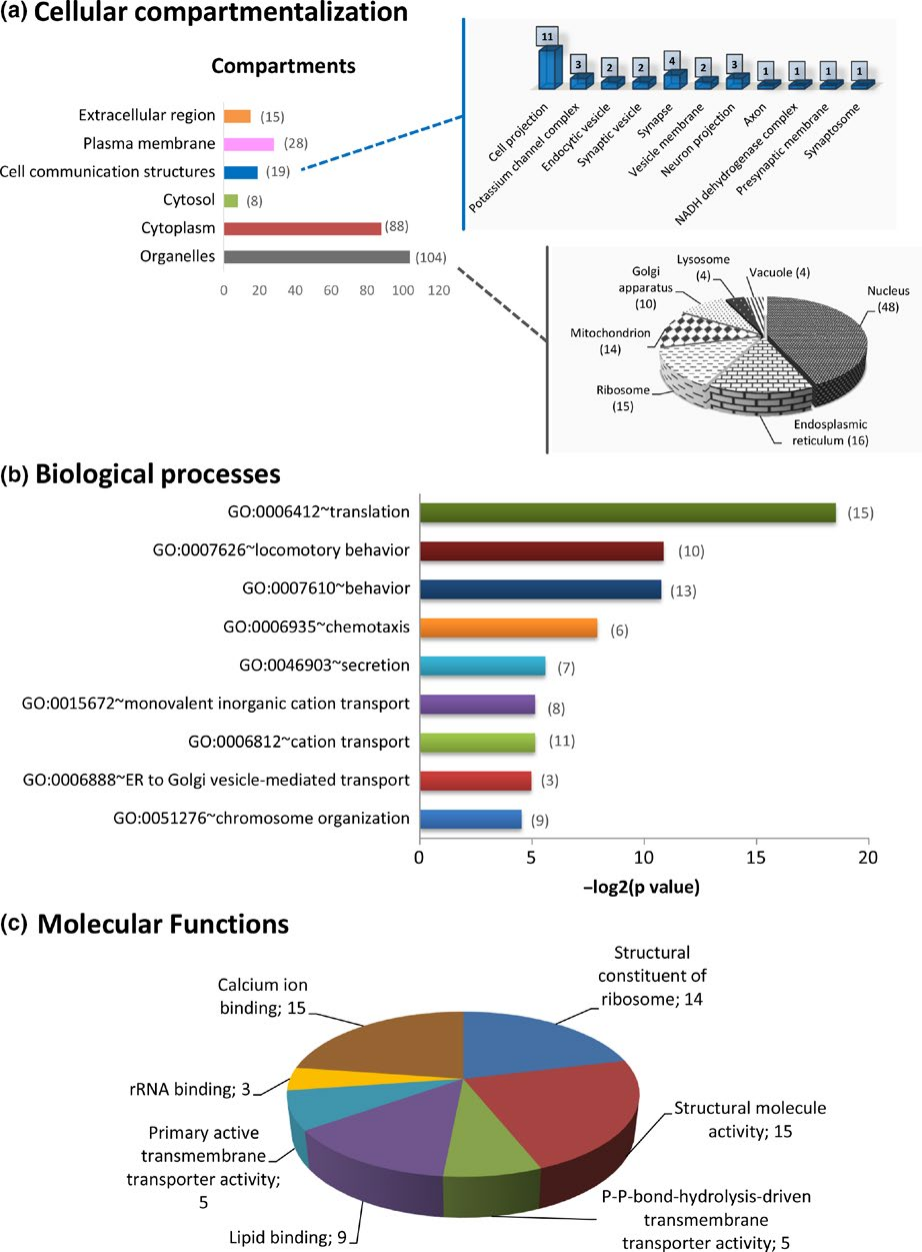
Gene ontology (GO) annotation and cluster classification analysis of mobile phone radiation DEGs in mouse hippocampus using the DAVID knowledgebase bioinformatics tools. DEGs were categorized according to (a) “cellular compartmentalization,” (b) “biological processes,” and (c) “molecular functions.” The number of genes that could be classified in each different category is indicated

DEGs were further analyzed through Gene Ontology (GO) and DAVID bioinformatics resources and classified according to their cellular topology, molecular function, and the biological processes of their respective cognate products (Figure 3). Functional annotation, based on DAVID database, regarding the cellular compartmentalization revealed that 15 proteins were localized in the extracellular region, whereas in the intracellular region, only 8 proteins were identified in the cytosol and 28 proteins were categorized as plasma membrane components. Interestingly, the majority of DEGs encoded proteins associated with organelles (104 proteins), mainly the nucleus (48 proteins). There were also gene products involved in the secretory network associated with organelles responsible for trafficking, that is, Golgi apparatus and endoplasmic reticulum, as well as with mitochondria and ribosomes (Figure 3a).

MP radiation‐specific DEGs were found to be involved in diverse biological processes, such as “translation” (15 proteins), “behavior” (13 proteins), “chemotaxis” (6 proteins), “secretion” (seven proteins), “cation transport” (11 proteins), “ER to Golgi vesicle‐mediated transport” (three proteins), and “chromosome organization” (nine proteins) (Figure 3b). The GO DAVID “Molecular Function” filter classified the DEGs into seven groups, namely “calcium ion binding” (15 proteins), “rRNA binding” (three proteins), “primary active transmembrane transporter activity” (five proteins), “lipid binding” (nine proteins), “structural constituent of ribosome” (14 proteins), “structural molecule activity” (15 proteins), and “P‐P‐bond‐hydrolysis‐driven transmembrane transporter activity (5 proteins) (Figure 3c).

#### 3.2.3 Molecular wiring of DEGs into canonical pathways and signaling networks

**Figure 4:**
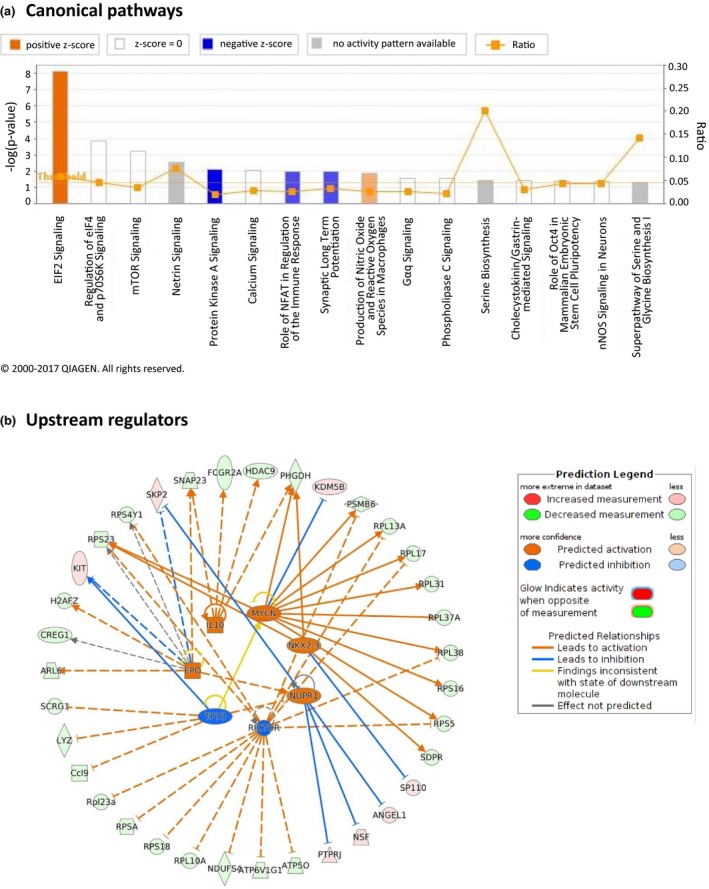
Canonical pathways analysis and identification of upstream regulators. (a) Bar graphs denote functional pathways that were significantly (*p *≤* *0.05) affected in hippocampus by exposure of mice to mobile phone radiation. Ratio is referred to the number of genes from our DEGs list divided by the total number of genes involved in each specific pathway that has been notably affected. Threshold is determined at *p* value 0.05 [−log (0.05)  = 1.30]. (b) Network of selected upstream transcription regulators, which were considered to be active or repressed regulating the expression of the genes affected by mobile phone radiation in mouse hippocampus, were merged in a single network

Pathway analysis identified 16 canonical pathways, which were significantly enriched (*p *≤* *0.05) with the 178 DEGs (Figure 4a), including “Calcium Signaling,” “Synaptic Long Term Potentiation,” “Production of Nitric Oxide and Reactive Oxygen Species in Macrophages,” “Serine Biosynthesis,” and “Signaling in Neurons.”

To gain further insight into the potential mechanisms of the effects of mobile phone radiation on the mouse brain, we were able to construct networks related to specific processes and diseases. Ten networks were identified and were ranked by the score in the *p* value calculation of the IPA assay, which ranged from 11 to 53 (Table 3). The highest‐scoring network revealed a significant link with “Cancer, Cell Death and Survival, Organismal Injury and Abnormalities.” Furthermore, annotation of the genes comprising the rest of the networks showed that most of the genes were mainly implicated in “Cellular Assembly and Organization, Cellular Function and Maintenance, Molecular Transport,” “Nucleic Acid Metabolism, Small Molecule Biochemistry, Cellular Function and Maintenance,” “Cancer, Protein Degradation, Protein Synthesis,” “Cell Cycle, Cancer, Cellular Development,” and in “Lipid Metabolism, Small Molecule Biochemistry, Molecular Transport” processes.

**Table 3:** Functional networks that were significantly affected by mobile phone radiation related to specific processes and diseases according to Ingenuity Pathway Analysis (IPA). Ten networks were identified and were ranked by the score in the *p* value calculation of the IPA assay, which ranged from 11 to 53. The higher the score number, the stronger the effect in the network. The components of these networks are shown in the “Molecules in Network” column, while the number of the genes of each network that were affected is shown in the “Focus molecules” column. The scores take into account the number of focus proteins and the size of the network to approximate the relevance of the network to the original list of focus proteins

ID	Molecules in network	Score	Focus molecules	Top diseases and functions
#1	60S ribosomal subunit,Akt,AQR,BCAM,CCDC90B,Cops2,CREG1,DHX8,EIF2S2,FRZB,IgG2a,Laminin,MYOC,Nfat (family), Pka catalytic subunit,PTN, Rbx1, Ribosomal 40s subunit, Rnr, RPL17, RPL31, RPL38, RPL10A, RPL13A, RPL37A, RPS5, RPS16, RPS18, RPS23, RPS4Y1, RPSA, SDC4, SKP2, TCEAL9, Ubiquitin	53	26	Cancer, Cell Death and Survival, Organismal Injury and Abnormalities
#2	ACP1, B4GALT6, BAIAP2, Ccl9, CDK14, Cg, DBI, DECR1, ENAH, EPHA4, EPM2A, ERK1/2, Fcer1, Growth hormone, HINT1, Ige, IGFBP6, Integrin, KIT, Lh, NSF, OVGP1, p85 (pik3r),PLC gamma, PSMB6, PTPase, PTPRJ, Rap1, RASGRP1, SNAP23, SRC (family), Syntaxin, TOPORS, USE1, VAMP7	42	22	Cellular Assembly and Organization, Cellular Function and Maintenance, Molecular Transport
#3	26s Proteasome, Actin, ANXA1, ATE1, Calcineurin protein(s), calpain, CAPN5, CD3, CORO1A, Creb, Cyclin A, F Actin, FCGR2A, GAS5, H2AFZ, HDAC9, HISTONE, Histone h3, Histone h4, HOPX, IKBKAP, IL12 (complex), Immunoglobulin, KDM5B, KDM5D, KIF5C, NCF2, NFkB (complex), PI3K (complex), Pkc(s), PPP3CC, PVALB, RNA polymerase II, TOB2, VPS35	32	18	Dermatological Diseases and Conditions, Gastrointestinal Disease, Immunological Disease
#4	ALB, AMN, ANGEL1, ATP2B2, Calmodulin, CELF2, DAPK2, DGKG, ERK, Focal adhesion kinase, Gsk3, HAUS5, IgG, IL1, Insulin, ITPR2, Jnk, LDL, LRRTM1, LYZ, Mapk, ODF2, P38 MAPK, PDE10A, PDGF BB, Pka, PLC, Rac, Ras, Ras homolog, RND2, RYR3, SYT1, Vegf, ZFYVE21	30	17	Nucleic Acid Metabolism, Small Molecule Biochemistry, Cellular Function and Maintenance
#5	APH1A, APP, ARF3, ATG12, ATP5O, ATP6V1G1, beta‐estradiol, C1D, C4A/C4B, CFB, CLUAP1, COG1, CSRP1, cyclic GMP, DECR1, dehydroepiandrosterone sulfate, DERL1, DKK1, EGFR, FSIP1, GNRH2, GRIN2A, GTF3C6, H + ‐transporting two‐sector ATPase, MBTPS1, PPP1R3C, PRSS3, RESP18, SEC61G, SUMO1, TAC1, TRAF3IP1, UBXN10, VARS, VHL	27	16	Cancer, Protein Degradation, Protein Synthesis
#6	AKR1A1, AMPD2, B3GALT1, BCO2, Brd4, CORO1B, DKK1, DMWD, EZH2, FETUB, HOXD12, HSPB1, JARID2, KRT5, Ku, LRRC40, MECOM, miR‐34a‐5p (and other miRNAs w/seed GGCAGUG), MRPL40, PGM2, PHGDH, PLEKHF1, POT1, PRKCDBP, RASGEF1C, Rian, Rpl23a, SACS, SDPR, SH3BP1, SHKBP1, SP110, TERF1, tretinoin, TRPC3	23	14	Cell Cycle, Cancer, Cellular Development
#7	ACADL, ARL16, BSCL2, CCL5, Ccl8, Ccl9, CCR10, Cd52, CFD, chemokine, CIDEA, CIDEC, CX3CL1, CXCL14, ENPP2, Ighd, IGHE, IGHM, KCTD14, LRRC20, miR‐1249‐5p (and other miRNAs w/seed GGAGGGA), NTAN1, PALB2, PLIN5, PNPLA2, PPARG, PPBP, RANBP3, RNase A, S100A8, S100a11, SLN, TAB1, THRSP, TMEM134	23	14	Lipid Metabolism, Small Molecule Biochemistry, Molecular Transport
#8	1,3,4,5‐IP4, Acox, anandamide, ARL6, Atp5k, CDK5R1, Cebp, CHGB, Ck2, CSNK2A1, DKK1, FGA, FHL3, FNBP4, Glycogen synthase, Gm561, GNA14, IFNG, KCNS2, L‐threonine, miR‐4651 (and other miRNAs w/seed GGGGUGG), POLR1A, PRKAR2A, PRNP, RNA polymerase I, RNASE4, SCP2, SERPING1, SLC34A2, SMARCD1, ST13, TAB1, TP53, TRUB2, ZFP36	17	11	Cell Cycle, Cellular Development, Embryonic Development
#9	ABCA1, ABCA8, APOC3, CDC45, DDX24, Delta/Jagged, DKK1, DLL3, farnesyl pyrophosphate, FASTKD2, FCGRT, HMG CoA synthase, Igkv1‐117, ILF3, LYL1, mevalonic acid, MIRLET7, MSGN1, MYC, NDUFS4, NFKBIA, NOTCH1, NPM1, NUP43, Pka catalytic subunit,PPARα‐RXRα, RNF20, RPP30, RPP40, SPOCK1, TAB1, TCP1, Tmsb4x (includes others), TRAF7, Zfp68	17	11	Lipid Metabolism, Molecular Transport, Small Molecule Biochemistry
#10	ACOT7, C1QB, CAPZA2, CCDC57, CD302, CKB, CLK3, EED, HSPH1, IKBKAP, JARID2, MIPOL1, miR‐16‐5p (and other miRNAs w/seed AGCAGCA), miR‐9‐3p (and other miRNAs w/seed UAAAGCU), MMD, MRPL20, MSH2, ORMDL1, PCDHA1, PCDHAC1, PLAG1, POLR2H, RBM4, RHOT1, SCRG1, SNRPA1, SPATA5, SREK1IP1, STAG1, VILL, XPO7, ZC3H11A, ZHX1, ZMAT2, ZMYM2	11	8	Cellular Compromise, Hematological System Development and Function, Auditory Disease

The fact that so many different functional pathways and networks were affected unveils the nontargeted and multilateral action of mobile phone radiation on the mouse brain.

#### 3.2.4 Mechanistic networks of DEGs upstream transcription regulators

Using IPA, we were able to identify seven upstream regulators that were significantly (*p* value ≤0.05) affected by mobile phone radiation, namely TP73 (tumor protein 73; inhibited), RICTOR (rapamycin‐insensitive companion of mammalian target of rapamycin; inhibited), NUPR1 (nuclear protein 1; activated), NKX2‐3 (homeobox protein Nkx2‐3; activated), MYCN (V‐Myc avian myelocytomatosis viral oncogene neuroblastoma‐derived homolog; activated), IL10 (Interleukin‐10; activated), and EPO (erythropoietin; activated), and we reconstructed a bigger mechanistic network including smaller networks with each one of the seven regulators and their cognate products (Figure 4b).

## 4 DISCUSSION

The impact of nonionizing radiofrequency electromagnetic radiation has attracted a lot of interest in recent years, because of the possible risks related to the chronic exposure of the human body. Importantly, the proposed effects of mobile phone radiation on the nervous, immune, and reproductive systems have been evaluated, suggesting oxidative stress and cell membrane gating and calcium oscillations as possible mechanisms of RF‐EMR action (Dasdag & Akdag, 2016; Desai, Kesari, & Agarwal, 2009; Manna & Ghosh, 2016; Minelli et al., 2007). Biomarkers are urgently needed to help us identify the potential mechanisms of RF‐EMR action and the effects on human pathophysiology. The omics approaches have been proposed as promising methods in this regard (Leszczynski & Joenväärä, 2001). Despite that numerous studies have been conducted to examine the ability of RF fields to affect a variety of cellular functions (Cotgreave, 2005), only a few studies have focused on the mobile phone radiation impact on brain transcriptome profile, while no study has been performed to analyze membrane phospholipid changes induced by MP or any other kind of RF radiation.

In the current work, after combining lipidomics and transcriptomics approaches, we demonstrated that MP radiation (SAR 0.022–0.366 W/kg), well below the permissible ICNIRP exposure limits for human‐head exposure (SAR 2 W/kg) (ICNIRP, 1998), but comparable to SAR levels produced in human brain regions (Kuster, Schuderer, Christ, Futter, & Ebert, 2004; Paparini et al., 2008), induces substantial phospholipid fatty acid remodeling in the brain, on the one hand, and on the other hand, it alters the expression of genes that are implicated in lipid metabolism.

The lipidomics analysis revealed that the mouse brain hippocampal tissue increased its MUFA content after whole‐body MP radiation exposure, whereas PUFA moieties were not significantly changed except for the level of one long‐chain omega‐3 PUFA, eicosapentaenoic acid EPA, which showed a significant decrease (cfr, Table 1). This is an intriguing effect of the exposure of the hippocampus, involving a specific omega‐3 fatty acid, such as EPA, that is known to be also a precursor for signaling lipids and gene transcription factors (Deckelbaum, Worgall, & Seo, 2006). Decreased levels of EPA are known to alter emotional behavior and memory in animal models (Bazinet & Laye, 2014), and also, n‐3 PUFAs deficiency alters hippocampal LTP, microglia activity, and brain plasticity in mice and is a causative factor of neurodevelopmental disorders (Madore et al., 2014; Thomazeau, Bosch‐Bouju, Manzoni, & Laye, 2017). We can hypothesize that the EPA decrease detected in our experiments could be related to the cognitive deficits shown previously by our group after mobile phone exposure (Fragopoulou et al., 2010; Ntzouni et al., 2011).

Using UI and PI indices (see Table 2), we could estimate the overall effects of the remodeling in terms of the total unsaturation indices which means the total contribution to biophysical properties and chemical reactivity of the tissue lipids, brought about by the mono‐ and poly‐unsaturated fatty acid residues. Fatty acid distribution among the different families during the radiation exposure showed a significant increase in MUFA compared to SFA. On the other hand, the EPA release discussed above did not have an effect on the total membrane unsaturation index. This is an important indication that a compensative effect between the increase in monounsaturated moieties and the decrease in polyunsaturated ones occurs. Further studies are needed in order to fully evaluate the meaning of this remodeling, especially considering the influence of dietary conditions, being polyunsaturated fatty acids connected with the intake of essential precursors from the diet.

Another interesting observation can be made with C16 MUFA residues, which are fatty acids negligibly present in foods (<0.1%), therefore connected with the enzymatic transformation of the common precursor, palmitic acid C16:0, by desaturase enzymatic activity. In fact, in our analysis, the position of the double bond was carefully examined, establishing that the delta‐9 desaturase product (palmitoleic acid 9cis‐16:1) was not increased, whereas sapienic acid increased, indicating a corresponding increase of delta‐6 desaturase activity in the processing of palmitic acid. Taking into account that delta‐6 desaturase is the enzyme preferentially processing PUFA (Sansone et al., 2016), the fact that in exposed mice, the delta‐6 metabolism increases for the SFA to MUFA transformation is intriguing. It can be observed that desaturation of fatty acids is a well‐known signal for tolerance in living organisms (Sakamoto & Murata, 2002), but no information on the positional isomers is available so far, although it is important to evaluate the lipid metabolism in detail. Considering linoleic acid (C18:2, omega‐6), which is the natural substrate of delta‐6 desaturase, in Table 1, this fatty acid decreased in treated animals, although not significantly. On the other hand, being EPA significantly reduced, which is involved in the cascade of delta 6 desaturase activity, this diminution could have had an effect on higher availability of the enzyme for palmitic acid. So far, there is a discussion in the literature whether the two PUFA pathways of omega‐6 and omega‐3 are competing for the same enzymes and at which extent, especially for delta‐6 desaturases which show also interesting variations connected to their isoforms (Hofacer et al., 2011; Lee, Lee, Kang, & Park, 2016; Park et al., 2016). In this study, we are inaugurating the hypothesis of a competition between delta‐9 and delta‐6 desaturase enzymes involved in the SFA and PUFA metabolic balance in exposed nervous cells. We found it interesting that the distinction of the positional C16 MUFA gives an important contribution to envisage metabolic differences, as shown here for MP radiation exposure.

Phospholipids are the main components of biological membranes that host several sensor systems and ion channels regulated by the fatty acid composition, using fluidity variations as an additional response and protection to environmental stress (Conde, Chaves, & Geros, 2011; Los & Murata, 2004). So far, there is evidence that ionizing radiation (IR) can affect membrane fatty acids and that membranes could serve as radiation sensors (Benderitter et al., 1999), but no evidence exists on the RF‐EMR action on membrane fatty acids. The interest was more toward oxidative injury and DNA damage, which are involved in the IR response (Kam & Banati, 2013). Some of us have previously shown that fatty acid changes are sensitive markers of radiation exposure in amphibians (Audette‐Stuart et al., 2012), whereas in trout exposed to food spiked with tritium, both liver and muscle tissues were found to change their fatty acids significantly compared to controls. Under these conditions, no significant differences in DNA strand break repair activity were found (Festarini et al., 2016). Those experiments showed that PUFA consumption was not occurring, but instead a remodeling response of the phospholipid fatty acid moieties with an increase in unsaturated components was taking place. This response can be indicated as the “first” sensitization targeting tissue lipids with an increase in unsaturation index, known in its turn to increase fluidity and permeability properties of membranes, as well as change of membrane protein and channel functioning (Maulucci et al., 2016), that could also be the case in our study following RF‐EMR exposure.

It is indeed interesting that fatty acid changes previously reported in tissues after *in vivo* radiation and tritium exposure of amphibians and fish also pointed out an increased MUFA content, thus suggesting that this aspect can be further developed as a specific radiation response (Audette‐Stuart et al., 2012; Festarini et al., 2016). Moreover, the present data on the EPA loss in the hippocampus of exposed mice suggest further development in view of the reported model of EPA supplementation that activated the interleukin‐1β‐induced cell signaling in the hippocampus of rats exposed to gamma irradiation (Lynch et al., 2003). Finally, the response of brain tissue to MP radiation suggests a possible role of nutritional status that provides unsaturated molecular components to tissues, which can favor or disfavor the correct body response to chronic radiation exposure.

Concerning the effects of MP radiation on the hippocampal transcriptome, we showed that the expression of 178 genes implicated in diverse cellular and molecular functions (lipid metabolism, calcium signaling, cell cycle regulation, cell death and survival, cancer, synaptic LTP, etc.) was altered 6–8 hr after whole‐body exposure, thus supporting the notion of a multifaceted impact of this kind of radiation on the murine brain. This notion is additionally supported by the fact that MP radiation had also a multitargeted effect onto subcellular organelles, as has also been previously shown by our group in Drosophila (Manta et al., 2017).

Importantly, the altered expression of genes implicated in the lipid metabolism may be associated with the changes that we observed on the fatty acid levels in the lipidomics analysis. Particularly, *Acadl*, the product of which catalyzes the initial step of mitochondrial beta‐oxidation of straight‐chain fatty acids, was found 1.83‐fold upregulated. The significance of such changes is valuable for elucidating the mechanism of action of the MP radiation, as it implicates that a specific lipid composition aggravates certain cell communication abnormalities caused by MP radiation. Studies showing hippocampal genome profile alterations associated with membrane functions (Nittby, Widegren, et al., 2008) and lipid peroxidation changes (Saikhedkar et al., 2014; Shahin, Singh, & Chaturvedi, 2017) following EMR exposure support our observations.

Furthermore, the fact that mechanisms such as LTP, calcium signaling, cell cycle regulation, and death were affected may be associated with the increased Ca^2+^ influx, apoptosis, synaptic changes, pyramidal cell loss, and reactive astrocytosis, which all have been reported to occur following MP radiation exposure (Bas, Odaci, Mollaoglu, Ucok, & Kaplan, 2009; Bas, Odaci, Kaplan, et al., 2009; Li et al., 2012; Maskey et al., 2010; Moghimi, Baharara, & Musavi, 2009; Narayanan et al., 2014; Odaci, Bas, & Kaplan, 2008; Sahin et al., 2015) and hence lead to learning and memory deficits, as previously reported by our group (Fragopoulou et al., 2010) and other groups (Li et al., 2012; Moghimi et al., 2009; Narayanan et al., 2015; Saikhedkar et al., 2014; Wang et al., 2013).

Interestingly, we also show that seven major gene regulators (TP73, RICTOR, NUPR1, NKX2‐3, MYCN, IL10, and EPO) involved in inflammation, oxidative stress, DNA damage, and cancer processes were affected by MP radiation. Specifically, the inhibition of TP73 and the activation of NUPR1 suggest that MP radiation may act as a stress and apoptotic factor. Stress and cell death induction has also been previously reported by our group in drosophila (Manta et al., 2017) and by other groups in the rat brain (Narayanan et al., 2014) and in human glioblastoma and other cancer cells (Chowdhury, Samant, Fodstad, & Shevde, 2009; Ratovitski, 2017). The inhibition of RICTOR, which is part of the mTOR pathway, reveals the MP radiation impact on cell growth and survival and on oxidative phosphorylation and other mitochondrial functions (Li, Long, He, Belshaw, & Scott, 2015). The activation of NKX2‐3 and MYCN, which are implicated in cell differentiation and oncogenesis (Kramer, Ribeiro, Arsenian‐Henriksson, Deller, & Rohrer, 2016; Nagel et al., 2017), could be associated with previously reported brain protein expression alterations related to neurogenesis and brain plasticity demonstrated by Salford’s group (Nittby, Grafstrom, et al., 2008) and our group (Fragopoulou et al., 2012), as well as with increased risk for brain tumor development (Hardell & Carlberg, 2009) following exposure to MP radiation. Finally, the activation of the last two regulators (IL‐10 and EPO), which are cytokines related to microglia and implicated in inflammatory processes, neurodegenerative disorders, brain injuries, and antiapoptotic functions (Lobo‐Silva, Carriche, Castro, Roque, & Saraiva, 2016; Tamura et al., 2017), may be related to the robust EPA decrease that we found and to the cognitive dysfunction previously reported by our group following MP radiation exposure (Fragopoulou et al., 2010).

Direct comparison with the few published studies so far is complicated because different brain tissues and exposure conditions, were used, nevertheless, some of the results are similar to ours. Belyaev and coworkers exposed rats to GSM 915 MHz radiation at whole‐body average SAR of 0.4 W/kg for 2 hr and analyzed the gene expression profile in cerebellum by Affymetrix U34 GeneChips representing 8800 rat genes (Belyaev et al., 2006). The results showed that 11 genes were upregulated in a range of 1.34–2.74 folds, and one gene was downregulated 0.48‐fold. The induced genes encoded proteins with diverse functions including neurotransmitter regulation, BBB, and melatonin production. Two years later, the same group (Nittby, Widegren, et al., 2008), in an attempt to explain their earlier observation of BBB leakage and albumin transport through brain capillaries in the rat cortex and hippocampus (Salford, Brun, Sturesson, Eberhardt, & Persson, 1994), showed that 6 hr exposure of rats to GSM 1800 MHz MP radiation at a whole‐body SAR value of 13 mW/kg, corresponding to a brain SAR value of 30 mW/kg, could alter Gene Ontology categories associated with membrane functions (extracellular region, signal transducer activity, intrinsic to membrane, and integral to membrane) 1 hr after the end of the exposure. On the other hand, using stringent constraints, Paparini and coworkers found no evidence of major transcriptional changes in the brains of mice exposed to 1800 MHz GSM signal for 1 hr at a whole body SAR of 1.1 W/kg and brain SAR of 0.2 W/kg (Paparini et al., 2008). McNamee and coworkers, after also using a high‐stringency statistical analysis approach [false discovery rate (FDR)‐adjusted for multiple comparisons], they did not find any convincing evidence of consistent changes in gene expression in any of the discrete mouse brain regions examined following short‐term (4 hr/day for 5 consecutive days) 1.9 GHz pulse‐modulated or continuous‐wave RF field exposure (SARs 0.2 and 1.4 W/kg).

A number of limitations must be taken into account when considering our findings. First, our experimental design (2 hr GSM 1800 mobile phone exposure, 4.3–17.5 V/m average field intensity, and tissue isolation 6 hr postexposure) was used in order to detect changes occurring after an intermediate duration recovery time following an acute exposure, given that the average time of gene transcription is 8 hr. Consequently, we have not detected the immediate changes or late changes. After our exploratory study, a more extensive analysis at different time‐points has to be performed in order to be able to answer the above issue, as has also been previously shown for ionizing radiation (Mahmoud‐Ahmed et al., 2006). Second, the small sample size of the arrays (*n *= 2 per group; each sample was a pool of 4 RNAs derived from 4 mice) and the lack of FDR correction of the microarray data does not allow any generalization of the gene expression results. However, the verification with qRT‐PCR of the expression levels of genes regulating different pathways and processes is the most robust indication that the microarrays data are reliable. Also, according to Kendziorski, Irizarry, Chen, Haag, and Gould (2005), pooling is recommended instead of individual sample processing, when fewer than three arrays are used in each condition (Kendziorski et al., 2005). Furthermore, pooled samples can mitigate the “noise” and/or “stochasticity” usually occurring in protein translation and lipid synthesis/catabolism, both reflecting variations in proteomic and lipidomic contents and landscapes among different animals. Additionally, they can normalize the SNP‐dependent effect in cellular functions and responses and as such can provide all examined mice with an “averaged” (“consensus”) type of transcriptional activity that now operates independently from the (“physiological”) mutational load (SNPs) of each mouse of the examined group. Lastly, our results are important in the search for biomarkers after acute RF‐EMR exposure, but should be complemented with assessment of changes following chronic exposure aiming to confirm or refute the epidemiological data of other groups showing increased risk of brain tumor development after 10 years of mobile phone or cordless phone use (Hardell & Carlberg, 2009).

Despite these limitations, our data provide first evidence that MP radiation alters the levels of several lipids and also the expression of a multitude of genes in mouse hippocampus and thus has a multitargeted impact. These molecular targets, revealed by omics approaches, may serve as biomarkers of MP radiation on the brain. MP radiation affects a wide range of critical biological processes linked to neurodegeneration, one of which being cellular membrane remodeling, and can elicit a number of neurological symptoms and disorders. Future studies of short‐term exposure with larger sample sizes are required to confirm and expand our preliminary findings, and also, long‐term exposure studies are needed to elucidate whether such changes may increase in the long run the susceptibility to neurodevelopmental and neurodegenrative disorders and even lead to brain tumor development.

## CONFLICT OF INTEREST

The authors report no conflict of interests. The authors alone are responsible for the content and writing of the manuscript.

## AUTHORS’ CONTRIBUTIONS

AFF, AP, M‐D P, AG, CF, DJS, DT, and LHM designed the experiments. AFF, EB, and NK performed animal welfare, euthanasia, and tissue isolation. AFF performed animal maintenance and exposure. AFF, KS, and LHM performed dosimetrical analysis. AFF, M‐D P, and AKM conducted microarray and qRT‐PCR experiments. AFF and AP were involved in bioinformatics and statistical microarray and qRT‐PCR data analysis. AS and CF conducted lipidomics experiments. AFF and AS performed lipidomics data statistical analysis. AFF and AP prepared microarray and qRT‐PCR figures and figure legends. AFF, AS, and CF prepared lipidomics figures and figure legends. AFF, M‐D P, AP, AKM, DJS, DT, and LHM were involved in microarray data interpretation. AFF, AS, CC, AG, CF, and LHM were involved in lipidomic data interpretation. CF, DT, and LHM provided reagents, consumables, and infrastructure. AFF and CF wrote the manuscript. All authors reviewed the manuscript according to their expertise and approved the manuscript in its final form.
