# Supplementary figures and images for: Hippocampal lipidome and transcriptome profile alterations triggered by acute exposure of mice to GSM 1800 MHz mobile phone radiation: An exploratory study
Source: Brain Behav. 2018 May 22;8(6):e01001. doi: 10.1002/brb3.1001 (PMC5991598; doi:10.1002/brb3.1001)

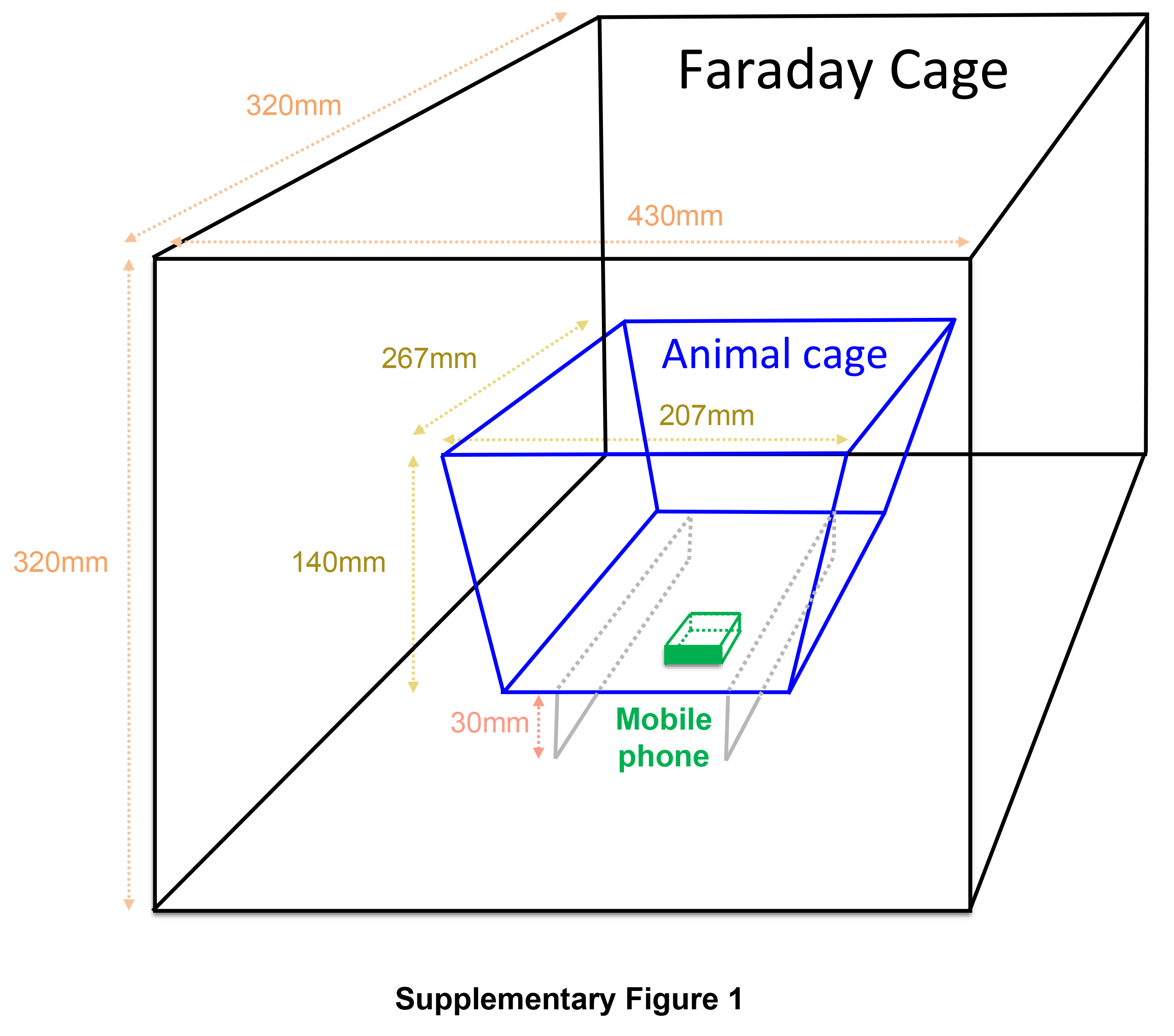

Supplement: Supplementary file 1 [file BRB3-8-e01001-s001.tif]

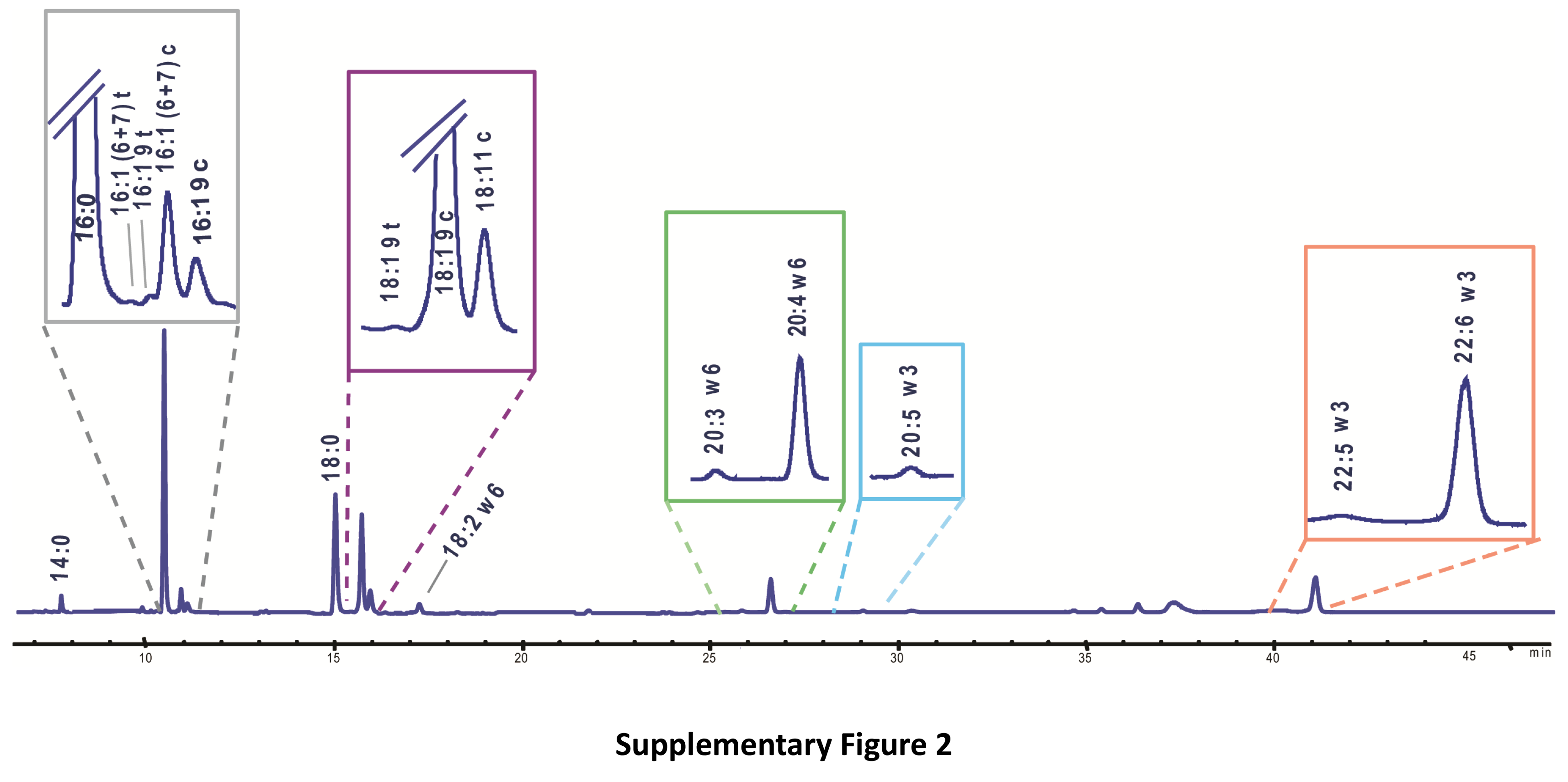

Supplement: Supplementary file 2 [file BRB3-8-e01001-s002.tif]

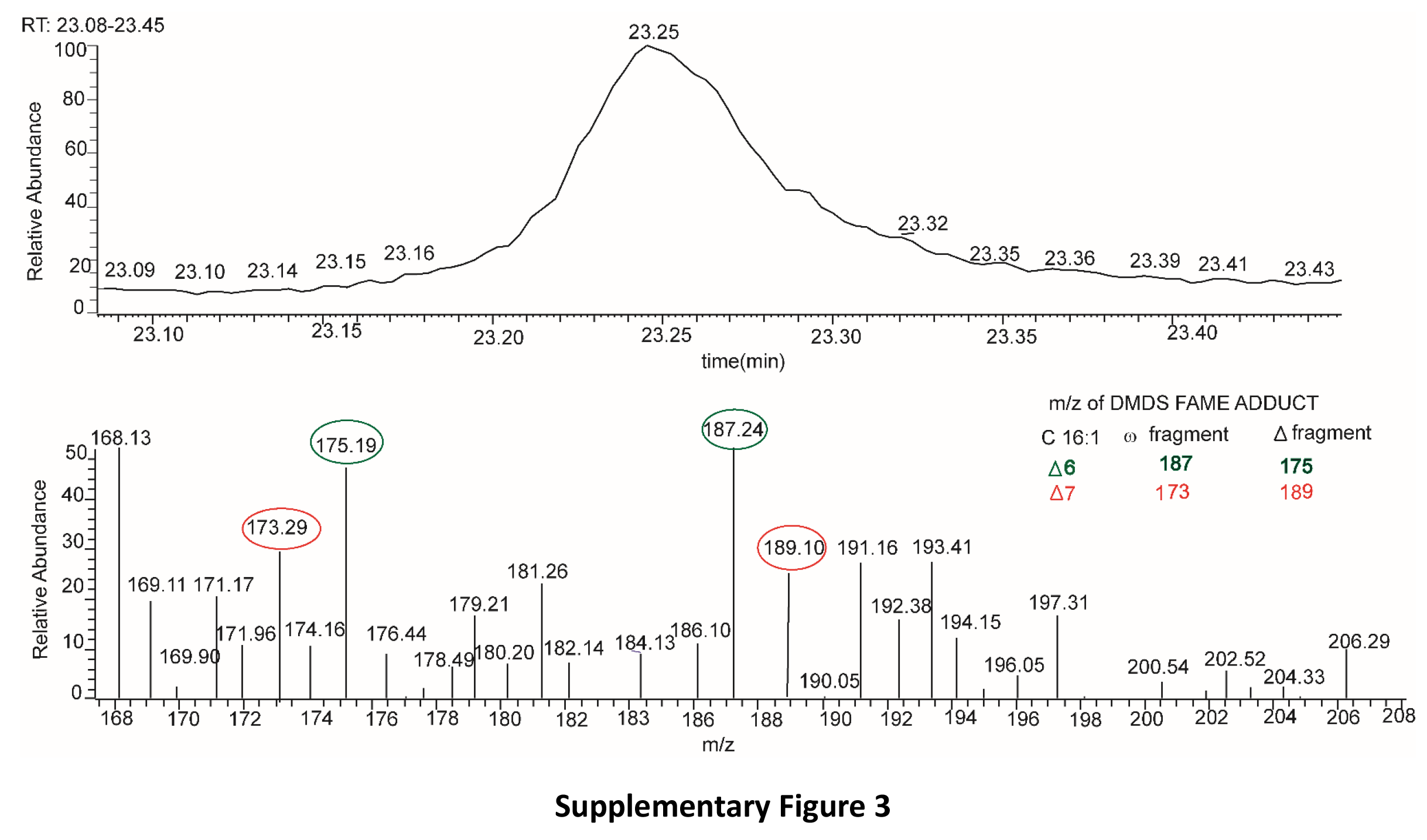

Supplement: Supplementary file 3 [file BRB3-8-e01001-s003.tif]
