# Supplementary material for: Hippocampal lipidome and transcriptome profile alterations triggered by acute exposure of mice to GSM 1800 MHz mobile phone radiation: An exploratory study
Source: Brain Behav. 2018 May 22;8(6):e01001. doi: 10.1002/brb3.1001 (PMC5991598; doi:10.1002/brb3.1001)
Supplement: Supplementary file 4 [file BRB3-8-e01001-s004.doc]

**Supplementary material**

**Supplementary Table 1:** Presentation of the name, oligonucleotide (forward and reverse) primer sequence, produced amplicon size of each one of the seven (including the *Gapdh* gene of reference) target genes, the expression levels of which were quantified herein by qRT-PCR technology.

| **Target gene name** | **Forward primer** | **Reverse primer** | **Amplicon size (bp)** |
| --- | --- | --- | --- |
| *Acad1* | 5’ GCTAATGCCTTACTTGGAGAAG 3’ | 5’ GTATGTGTGCAACTGTTTTCCC 3’ | 184 |
| *Alb* | 5’ CCCAATGCTGACTTTGCAG 3’ | 5’ TGCAGTTTGCTGGAGATAGTC 3’ | 159 |
| *Coro1a* | 5’ CCATGACAGTGCCTAGAAAG 3’ | 5’ CCATCCTTGAGGGAAATGAG 3’ | 139 |
| *Ear3* | 5’ TCCCGACTTTGTCTCCTGC 3’ | 5’ TATGCTGGATGGCAAACCACC 3’ | 93 |
| *Gapdh* | 5’ AGGTCGGTGTGAACGGATTTG 3’ | 5’ TGTAGACCATGTAGTTGAGGTCA 3’ | 122 |
| *Itpr2* | 5’ GACACCAAGCTGAAGGTTATC 3’ | 5’ GTCGTTGTTGTCTCCAAATTCC 3’ | 114 |
| *Terf1* | 5’ TCAGTAGGAACGAAGCAAGAAC 3’ | 5’ CATCAGTGTCTGGCTGATTC 3’ | 117 |
