# Supplementary material for: Hippocampal lipidome and transcriptome profile alterations triggered by acute exposure of mice to GSM 1800 MHz mobile phone radiation: An exploratory study
Source: Brain Behav. 2018 May 22;8(6):e01001. doi: 10.1002/brb3.1001 (PMC5991598; doi:10.1002/brb3.1001)
Supplement: Supplementary file 5 [file BRB3-8-e01001-s005.doc]

**Supplementary Table 2:** Table of the differentially expressed genes identified from microarray data. In the table we present the gene name, gene description, entrez gene id, fold change and p-value calculated from Welch’s t-test. Down-regulated genes in the exposed group are noted with blue color while up-regulated genes are noted with red color.

| **GeneSymbol** | **Gene Title** | **ENTREZ_**  **GENE_ID** | **fold** | **P value Welch** |
| --- | --- | --- | --- | --- |
| Coro1a | coronin, actin binding protein 1A | 12721 | 0,035 | 0,024 |
| Pde10a | phosphodiesterase 10A | 23984 | 0,049 | 0,035 |
| Dapk2 | death-associated protein kinase 2 | 13143 | 0,212 | 0,040 |
| Gna14 | guanine nucleotide binding protein, alpha 14 | 14675 | 0,232 | 0,021 |
| Enpp2 | ectonucleotide pyrophosphatase/phosphodiesterase 2 | 18606 | 0,283 | 0,011 |
| Obfc2a | oligonucleotide/oligosaccharide-binding fold containing 2A | 109019 | 0,332 | 0,046 |
| Frzb | frizzled-related protein | 20378 | 0,348 | 0,031 |
| Brd4 | bromodomain containing 4 | 57261 | 0,353 | 0,003 |
| Rasgrp1 | RAS guanyl releasing protein 1 | 19419 | 0,387 | 0,028 |
| Epha4 | Eph receptor A4 | 13838 | 0,395 | 0,020 |
| Sp110 | Sp110 nuclear body protein | 109032 | 0,413 | 0,027 |
| Pcdhac1 | protocadherin alpha subfamily C, 1 | 353236 | 0,424 | 0,045 |
| Terf1 | telomeric repeat binding factor 1 | 21749 | 0,439 | 0,006 |
| Skp2 | S-phase kinase-associated protein 2 (p45) | 27401 | 0,444 | 0,046 |
| Mbtps1 | membrane-bound transcription factor peptidase, site 1 | 56453 | 0,468 | 0,037 |
| Ovgp1 | oviductal glycoprotein 1 | 12659 | 0,477 | 0,011 |
| Aqr | aquarius | 11834 | 0,479 | 0,034 |
| Ate1 | arginyltransferase 1 | 11907 | 0,485 | 0,020 |
| Kdm5b | lysine (K)-specific demethylase 5B | 75605 | 0,522 | 0,030 |
| Ryr3 | ryanodine receptor 3 | 20192 | 0,525 | 0,033 |
| Ikbkap | inhibitor of kappa light polypeptide enhancer in B-cells, kinase complex-associated protein | 230233 | 0,526 | 0,020 |
| Celf2 | CUGBP, Elav-like family member 2 | 14007 | 0,542 | 0,039 |
| Nsf | N-ethylmaleimide sensitive fusion protein | 18195 | 0,548 | 0,032 |
| Abca8b | ATP-binding cassette, sub-family A (ABC1), member 8b | 27404 | 0,550 | 0,010 |
| Slc34a2 | solute carrier family 34 (sodium phosphate), member 2 | 20531 | 0,554 | 0,028 |
| Polr1a | polymerase (RNA) I polypeptide A | 20019 | 0,557 | 0,035 |
| Kit | kit oncogene | 16590 | 0,558 | 0,048 |
| Lrrtm1 | leucine rich repeat transmembrane neuronal 1 | 74342 | 0,567 | 0,011 |
| Fnbp4 | formin binding protein 4 | 55935 | 0,574 | 0,044 |
| Mll1 | myeloid/lymphoid or mixed-lineage leukemia 1 | 214162 | 0,577 | 0,044 |
| Ampd2 | adenosine monophosphate deaminase 2 | 109674 | 0,582 | 0,023 |
| Dll3 | delta-like 3 (Drosophila) | 13389 | 0,588 | 0,049 |
| Topors | topoisomerase I binding, arginine/serine-rich | 106021 | 0,593 | 0,009 |
| 4930432F04Rik | RIKEN cDNA 4930432F04 gene | 78792 | 0,593 | 0,018 |
| Large | like-glycosyltransferase | 16795 | 0,596 | 0,019 |
| Dgkg | diacylglycerol kinase, gamma | 110197 | 0,597 | 0,032 |
| Arf3 | ADP-ribosylation factor 3 | 11842 | 0,600 | 0,044 |
| Pcdha1 | protocadherin alpha 1 /// protocadherin alpha 10 /// protocadherin alpha 11 /// protocadherin alpha 12 /// protocadherin alpha 2 /// protocadherin alpha 3 /// protocadherin alpha 4 /// protocadherin alpha 5 /// protocadherin alpha 6 /// protocadherin alpha 7 /// protocadherin alpha 8 /// protocadherin alpha 9 /// protocadherin alpha subfamily C, 1 /// protocadherin alpha subfamily C, 2 | 116731 /// 12936 /// 12937 /// 12939 /// 12941 /// 12942 /// 12943 /// 192161 /// 192163 /// 192164 /// 353234 /// 353235 /// 353236 /// 353237 | 0,601 | 0,041 |
| Kcns2 | K+ voltage-gated channel, subfamily S, 2 | 16539 | 0,604 | 0,023 |
| Spock1 | sparc/osteonectin, cwcv and kazal-like domains proteoglycan 1 | 20745 | 0,605 | 0,014 |
| Dhx8 | DEAH (Asp-Glu-Ala-His) box polypeptide 8 | 217207 | 0,609 | 0,024 |
| B4galt6 | UDP-Gal:betaGlcNAc beta 1,4-galactosyltransferase, polypeptide 6 | 56386 | 0,612 | 0,044 |
| Capn5 | calpain 5 | 12337 | 0,615 | 0,022 |
| Angel1 | angel homolog 1 (Drosophila) | 68737 | 0,615 | 0,031 |
| Syt1 | synaptotagmin I | 20979 | 0,622 | 0,035 |
| Odf2 | outer dense fiber of sperm tails 2 | 18286 | 0,627 | 0,032 |
| Lrrc20 | leucine rich repeat containing 20 | 216011 | 0,629 | 0,043 |
| Cdk14 | cyclin-dependent kinase 14 | 18647 | 0,642 | 0,026 |
| Rnf20 | ring finger protein 20 | 109331 | 0,642 | 0,043 |
| Bscl2 | Bernardinelli-Seip congenital lipodystrophy 2 homolog (human) | 14705 | 0,648 | 0,007 |
| Baiap2 | brain-specific angiogenesis inhibitor 1-associated protein 2 | 108100 | 0,648 | 0,049 |
| Ptprj | protein tyrosine phosphatase, receptor type, J | 19271 | 0,650 | 0,029 |
| Vill | villin-like | 22351 | 0,652 | 0,032 |
| C630004H02Rik | RIKEN cDNA C630004H02 gene | 217310 | 0,653 | 0,009 |
| Ilf3 | interleukin enhancer binding factor 3 | 16201 | 0,654 | 0,030 |
| Haus5 | HAUS augmin-like complex, subunit 5 | 71909 | 0,658 | 0,038 |
| Cog1 | component of oligomeric golgi complex 1 /// conserved oligomeric Golgi complex subunit 1-like | 100504959 /// 16834 | 0,658 | 0,048 |
| Kif5c | kinesin family member 5C | 16574 | 0,660 | 0,030 |
| Ppp3cc | protein phosphatase 3, catalytic subunit, gamma isoform | 19057 | 0,661 | 0,044 |
| Atp2b2 | ATPase, Ca++ transporting, plasma membrane 2 | 11941 | 0,663 | 0,010 |
| Atg12 | autophagy-related 12 (yeast) | 67526 | 1,501 | 0,042 |
| Ccl9 | chemokine (C-C motif) ligand 9 | 20308 | 1,504 | 0,003 |
| Rg9mtd1 | RNA (guanine-9-) methyltransferase domain containing 1 | 52575 | 1,507 | 0,035 |
| Sln | sarcolipin | 66402 | 1,521 | 0,043 |
| Rian | RNA imprinted and accumulated in nucleus | 75745 | 1,523 | 0,018 |
| D0H4S114 | DNA segment, human D4S114 | 27528 | 1,524 | 0,049 |
| Ptn | pleiotrophin | 19242 | 1,529 | 0,010 |
| Ccdc90b | coiled-coil domain containing 90B | 66365 | 1,531 | 0,037 |
| Eif2s2 | eukaryotic translation initiation factor 2, subunit 2 (beta) | 67204 | 1,533 | 0,041 |
| Vps35 | vacuolar protein sorting 35 | 65114 | 1,534 | 0,045 |
| Enah | enabled homolog (Drosophila) | 13800 | 1,538 | 0,004 |
| Ntan1 | N-terminal Asn amidase | 18203 | 1,538 | 0,034 |
| B3galt1 | UDP-Gal:betaGlcNAc beta 1,3-galactosyltransferase, polypeptide 1 | 26877 | 1,541 | 0,005 |
| Rnd2 | Rho family GTPase 2 | 11858 | 1,543 | 0,009 |
| Atp5o | ATP synthase, H+ transporting, mitochondrial F1 complex, O subunit | 28080 | 1,546 | 0,024 |
| Resp18 | regulated endocrine-specific protein 18 | 19711 | 1,551 | 0,032 |
| Tmem134 | transmembrane protein 134 | 66990 | 1,557 | 0,039 |
| Sdpr | serum deprivation response | 20324 | 1,569 | 0,024 |
| Zfyve21 | zinc finger, FYVE domain containing 21 | 68520 | 1,571 | 0,029 |
| S100a11 | S100 calcium binding protein A11 (calgizzarin) | 20195 | 1,577 | 0,041 |
| Kdm5d | lysine (K)-specific demethylase 5D | 20592 | 1,586 | 0,014 |
| Acp1 | acid phosphatase 1, soluble | 11431 | 1,594 | 0,017 |
| Trub2 | TruB pseudouridine (psi) synthase homolog 2 (E. coli) | 227682 | 1,599 | 0,026 |
| Gtf3c6 | general transcription factor IIIC, polypeptide 6, alpha | 67371 | 1,610 | 0,027 |
| 1600012F09Rik | RIKEN cDNA 1600012F09 gene | 67008 | 1,612 | 0,026 |
| Rpl10a | ribosomal protein L10A | 19896 | 1,615 | 0,033 |
| Zmat2 | zinc finger, matrin type 2 | 66492 | 1,616 | 0,021 |
| Bco2 | beta-carotene oxygenase 2 | 170752 | 1,616 | 0,046 |
| Plekhf1 | pleckstrin homology domain containing, family F (with FYVE domain) member 1 | 72287 | 1,617 | 0,042 |
| Gas5 | growth arrest specific 5 | 14455 | 1,622 | 0,038 |
| Fastkd2 | FAST kinase domains 2 | 75619 | 1,623 | 0,038 |
| Lyl1 | lymphoblastomic leukemia 1 | 17095 | 1,624 | 0,017 |
| Sdc4 | syndecan 4 | 20971 | 1,627 | 0,033 |
| Use1 | unconventional SNARE in the ER 1 homolog (S. cerevisiae) | 67023 | 1,629 | 0,034 |
| Cops2 | COP9 (constitutive photomorphogenic) homolog, subunit 2 (Arabidopsis thaliana) | 12848 | 1,629 | 0,030 |
| Cxcl14 | chemokine (C-X-C motif) ligand 14 | 57266 | 1,634 | 0,046 |
| Rps4x | ribosomal protein S4, X-linked | 20102 | 1,637 | 0,033 |
| Arl6 | ADP-ribosylation factor-like 6 | 56297 | 1,642 | 0,046 |
| Gm14430 | predicted gene 14430 /// predicted gene 14434 | 627914 /// 668039 | 1,648 | 0,050 |
| Igfbp6 | insulin-like growth factor binding protein 6 | 16012 | 1,652 | 0,023 |
| Myoc | myocilin | 17926 | 1,656 | 0,038 |
| 0610009B22Rik | RIKEN cDNA 0610009B22 gene | 66050 | 1,661 | 0,044 |
| Cd52 | CD52 antigen | 23833 | 1,665 | 0,036 |
| Zfp68 | zinc finger protein 68 | 24135 | 1,667 | 0,041 |
| Atp6v1g1 | ATPase, H+ transporting, lysosomal V1 subunit G1 | 66290 | 1,669 | 0,033 |
| Decr1 | 2,4-dienoyl CoA reductase 1, mitochondrial | 67460 | 1,674 | 0,016 |
| Ncf2 | neutrophil cytosolic factor 2 | 17970 | 1,676 | 0,033 |
| Rpl23a | ribosomal protein L23a | 268449 | 1,676 | 0,048 |
| 3200002M19Rik | RIKEN cDNA 3200002M19 gene | 75430 | 1,677 | 0,013 |
| Sepp1 | selenoprotein P, plasma, 1 | 20363 | 1,682 | 0,041 |
| Wbp5 | WW domain binding protein 5 | 22381 | 1,684 | 0,045 |
| Phgdh | 3-phosphoglycerate dehydrogenase | 236539 | 1,691 | 0,028 |
| Gm561 | predicted gene 561 | 228715 | 1,696 | 0,021 |
| Rpl31 | ribosomal protein L31 | 114641 | 1,707 | 0,049 |
| LOC100045866 | transcription elongation factor B polypeptide 1-like /// transcription elongation factor B (SIII), polypeptide 1 | 100045866 /// 67923 | 1,716 | 0,045 |
| Rps23 | ribosomal protein S23 | 66475 | 1,720 | 0,030 |
| Hopx | HOP homeobox | 74318 | 1,724 | 0,039 |
| Rpl38 | ribosomal protein L38 | 67671 | 1,737 | 0,035 |
| Zfp759 | zinc finger protein 759 | 268670 | 1,746 | 0,039 |
| Shkbp1 | Sh3kbp1 binding protein 1 | 192192 | 1,747 | 0,011 |
| Rpsa | ribosomal protein SA | 16785 | 1,747 | 0,049 |
| Prkcdbp | protein kinase C, delta binding protein | 109042 | 1,748 | 0,004 |
| Gm12191 | ribosomal protein L30 pseudogene /// ribosomal protein L30 | 19946 /// 666899 | 1,748 | 0,036 |
| Scp2 | sterol carrier protein 2, liver | 20280 | 1,755 | 0,032 |
| Rnase4 | ribonuclease, RNase A family 4 | 58809 | 1,773 | 0,026 |
| Cd302 | CD302 antigen | 66205 | 1,780 | 0,007 |
| Hint1 | histidine triad nucleotide binding protein 1 | 15254 | 1,785 | 0,038 |
| Lyz1 | lysozyme 1 | 17110 | 1,794 | 0,037 |
| Gm11275 | predicted gene 11275 /// histone cluster 1, H4a /// histone cluster 1, H4b /// histone cluster 1, H4c /// histone cluster 1, H4h /// histone cluster 1, H4i /// histone cluster 1, H4j /// histone cluster 1, H4k /// histone cluster 1, H4m | 100041230 /// 319155 /// 319158 /// 319159 /// 319160 /// 319161 /// 326619 /// 326620 /// 69386 | 1,794 | 0,046 |
| Ccdc23 | coiled-coil domain containing 23 | 69216 | 1,794 | 0,029 |
| Fcgr3 | Fc receptor, IgG, low affinity III | 14131 | 1,802 | 0,029 |
| Rps16 | ribosomal protein S16 /// ribosomal protein S16, pseudogene 2 | 100039355 /// 20055 | 1,804 | 0,035 |
| Psmb6 | proteasome (prosome, macropain) subunit, beta type 6 | 19175 | 1,806 | 0,036 |
| Phospho2 | phosphatase, orphan 2 | 73373 | 1,821 | 0,008 |
| Ppp1r3c | protein phosphatase 1, regulatory (inhibitor) subunit 3C | 53412 | 1,825 | 0,042 |
| Pvalb | parvalbumin | 19293 | 1,836 | 0,042 |
| Scrg1 | scrapie responsive gene 1 | 20284 | 1,841 | 0,025 |
| Mrpl40 | mitochondrial ribosomal protein L40 | 18100 | 1,851 | 0,041 |
| Sumo1 | SMT3 suppressor of mif two 3 homolog 1 (yeast) | 22218 | 1,856 | 0,042 |
| Snap23 | synaptosomal-associated protein 23 | 20619 | 1,859 | 0,042 |
| Rbx1 | ring-box 1 | 56438 | 1,861 | 0,036 |
| Ndufs4 | NADH dehydrogenase (ubiquinone) Fe-S protein 4 | 17993 | 1,863 | 0,050 |
| Kctd14 | potassium channel tetramerisation domain containing 14 | 233529 | 1,884 | 0,012 |
| Nup43 | nucleoporin 43 | 69912 | 1,909 | 0,041 |
| Creg1 | cellular repressor of E1A-stimulated genes 1 | 433375 | 1,915 | 0,026 |
| Ubxn10 | UBX domain protein 10 | 212190 | 1,917 | 0,002 |
| Rps5 | ribosomal protein S5 | 20103 | 1,917 | 0,026 |
| C1d | C1D nuclear receptor co-repressor | 57316 | 1,920 | 0,045 |
| Rpl17 | ribosomal protein L17 | 319195 | 1,939 | 0,010 |
| Amn | amnionless | 93835 | 1,943 | 0,027 |
| Rpl13a | ribosomal protein L13A | 22121 | 1,945 | 0,048 |
| Csrp1 | cysteine and glycine-rich protein 1 | 13007 | 1,952 | 0,018 |
| Fsip1 | fibrous sheath-interacting protein 1 | 71313 | 1,969 | 0,040 |
| Atp5k | ATP synthase, H+ transporting, mitochondrial F1F0 complex, subunit e | 11958 | 1,995 | 0,010 |
| Thrsp | thyroid hormone responsive SPOT14 homolog (Rattus) | 21835 | 2,006 | 0,033 |
| Rpl37a | ribosomal protein L37a | 19981 | 2,012 | 0,030 |
| Tob2 | transducer of ERBB2, 2 | 57259 | 2,057 | 0,030 |
| Gm10393 | predicted gene 10393 /// placenta specific 9 | 100039246 /// 211623 | 2,064 | 0,028 |
| Gm10166 | predicted pseudogene 10166 /// predicted pseudogene 10294 /// ribosomal protein L17 /// ribosomal protein L17, pseudogene 3 | 100040745 /// 100042880 /// 100043872 /// 319195 | 2,065 | 0,005 |
| Ormdl1 | ORM1-like 1 (S. cerevisiae) | 227102 | 2,075 | 0,012 |
| Epm2a | epilepsy, progressive myoclonic epilepsy, type 2 gene alpha | 13853 | 2,082 | 0,041 |
| Dbi | diazepam binding inhibitor | 13167 | 2,102 | 0,036 |
| 5830411J07Rik | RIKEN cDNA 5830411J07 gene | 74742 | 2,107 | 0,014 |
| 0610037P05Rik | RIKEN cDNA 0610037P05 gene | 66086 | 2,139 | 0,041 |
| Vamp7 | vesicle-associated membrane protein 7 | 20955 | 2,152 | 0,026 |
| Rpp40 | ribonuclease P 40 subunit (human) | 208366 | 2,155 | 0,026 |
| Rps18 | ribosomal protein S18 | 20084 | 2,155 | 0,028 |
| H2afz | H2A histone family, member Z /// hypothetical LOC100504949 | 100504949 /// 51788 | 2,187 | 0,001 |
| Sec61g | SEC61, gamma subunit | 20335 | 2,244 | 0,027 |
| BC013712 | cDNA sequence BC013712 | 230787 | 2,340 | 0,023 |
| Anxa1 | annexin A1 | 16952 | 2,662 | 0,025 |
| Bcam | basal cell adhesion molecule | 57278 | 2,678 | 0,010 |
| Hdac9 | histone deacetylase 9 | 79221 | 2,818 | 0,036 |
| Arl16 | ADP-ribosylation factor-like 16 | 70317 | 3,523 | 0,041 |
| Alb | albumin | 11657 | 5,663 | 0,044 |
| Itpr2 | inositol 1,4,5-triphosphate receptor 2 | 16439 | 6,593 | 0,019 |
| Acadl | acyl-Coenzyme A dehydrogenase, long-chain | 11363 | 7,736 | 0,014 |
| Ear3 | eosinophil-associated, ribonuclease A family, member 3 | 53876 | 43,637 | 0,031 |
