# Supplementary material for: Hippocampal lipidome and transcriptome profile alterations triggered by acute exposure of mice to GSM 1800 MHz mobile phone radiation: An exploratory study
Source: Brain Behav. 2018 May 22;8(6):e01001. doi: 10.1002/brb3.1001 (PMC5991598; doi:10.1002/brb3.1001)
Supplement: Supplementary file 6 [file BRB3-8-e01001-s006.docx]

**Supplementary Table 3:** Table presenting average (AVG) normalized mRNA levels ± SD and *p* values calculated from Welch’s t-test (Exp1 and Exp2 vs. SE1 and SE2) after qRT-PCR analysis of selected genes.

| **Gene symbol** | **Sham-exposed** | | **Exposed** | | **Welch t-test p value** |
| --- | --- | --- | --- | --- | --- |
|  | AVG | SD | AVG | SD |  |
| *AcadI* | 336,62 | 94,77 | 612,76 | 156,60 | 0,001 |
| *Alb* | 0,66 | 0,29 | 1,55 | 0,57 | 0,003 |
| *Ear3* | 0,59 | 0,19 | 1,54 | 0,39 | <0,001 |
| *Itpr2* | 3,84 | 1,09 | 7,31 | 1,54 | 0,017 |
| *Coro1* | 338,31 | 86,59 | 223,39 | 65,57 | 0,017 |
| *Terf1* | 94,01 | 84,24 | 40,36 | 21,37 | 0,049 |
